# Prevalence and Genetic Diversity of Staphylococcal Enterotoxin (-Like) Genes *sey*, *selw*, *selx*, *selz*, *sel26* and *sel27* in Community-Acquired Methicillin-Resistant *Staphylococcus aureus*

**DOI:** 10.3390/toxins12050347

**Published:** 2020-05-23

**Authors:** Meiji Soe Aung, Noriko Urushibara, Mitsuyo Kawaguchiya, Masahiko Ito, Satoshi Habadera, Nobumichi Kobayashi

**Affiliations:** 1 Department of Hygiene, Sapporo Medical University School of Medicine, Sapporo 060-8556, Japan; noriko-u@sapmed.ac.jp (N.U.); kawaguchiya@sapmed.ac.jp (M.K.); nkobayas@sapmed.ac.jp (N.K.); 2 Sapporo Clinical Laboratory, Inc., Sapporo 060-0005, Japan; m-ito@saturin.co.jp (M.I.); s-habadera@saturin.co.jp (S.H.)

**Keywords:** *Staphylococcus aureus*, enterotoxin, *sey*, *selw*, *selx*, *selz*

## Abstract

Staphylococcal enterotoxins (SEs) are virulence factors of *Staphylococcus aureus* associated with various toxic diseases due to their emetic and superantigenic activities. Although at least 27 SE(-like) genes have been identified in *S. aureus* to date, the newly identified SE(-like) genes have not yet been well characterized by their epidemiological features. In this study, the prevalence and genetic diversity of SE gene *sey* and SE-like genes *selw*, *selx*, *selz*, *sel26*, and *sel27* were investigated for 624 clinical isolates of community-acquired methicillin-resistant *S. aureus* (CA-MRSA). The most prevalent SE(-like) gene was *selw* (92.9%), followed by *selx* (85.6%), *sey* (35.4%) and *selz* (5.6%), while *sel26* and *sel27* were not detected. Phylogenetically, *sey*, *selw*, *selx*, and *selz* were discriminated into 7, 10, 16, and 9 subtypes (groups), respectively. Among these subtypes, *sey* was the most conserved and showed the highest sequence identity (>98.8%), followed by *selz* and *selx*. The SE-like gene *selw* was the most divergent, and four out of ten genetic groups contained pseudogenes that may encode truncated product. Individual subtypes of SE(-like) genes were generally found in isolates with specific genotypes/lineages of *S. aureus*. This study revealed the putative ubiquity of *selw* and *selx* and the prevalence of *sey* and *selz* in some specific lineages (e.g., ST121) in CA-MRSA, suggesting a potential role of these newly described SEs(-like) in pathogenicity.

## 1. Introduction

*Staphylococcus aureus* is one of the most common pathogens in humans and is responsible for various diseases ranging from skin and soft tissue infections to severe and often deadly infections such as bacteremia [1]. Clinical isolates of *S. aureus* have been distinguished between methicillin-susceptible and -resistant *S. aureus* (MSSA and MRSA, respectively) based on the presence of the *mecA* gene associated with resistance to beta-lactam antibiotics. While healthcare-associated MRSA (HA-MRSA) was initially recognized as a major nosocomial pathogen worldwide, the emergence and spread of community-associated MRSA (CA-MRSA) since the 1990s has been a global public health concern until today [2].

A group of superantigens, i.e., staphylococcal enterotoxins (SEs) and toxic shock syndrome toxin-1 (TSST-1), is produced by most clinical isolates of *S. aureus* as etiological factors of toxic diseases including food poisoning and toxic shock syndrome [3]. To date, at least 27 SE or SE-like proteins have been identified, and most of them (SEA-SEE, SEG-SEI, SEK-SET, SEY) were demonstrated to have emetic activity in animals [3,4,5]. SElJ is a non-emetic protein, as well as TSST-1, while the emetogenicity of remaining SE-like proteins is yet to be determined. Bacterial superantigens that are produced by staphylococci and streptococci are phylogenetically classified into five groups, among which four groups (I, II, III, and V) include SEs and TSST-1 [3,4,6]. The prevalence of SE genes including *sea*-*see* and *seg*-*selu* in *S. aureus* has been analyzed in many studies of isolates from bacteremia [7,8], diabetic foot ulcers [9], cystic fibrosis [10], and colonization in healthy humans [11,12], as well as those from animals and the environment [13,14,15]. Although the distribution of SE genes *sea*-*see* and *seg*-*seo* (or -*seu*) to clinical isolates of HA- and CA-MRSA was also investigated previously [16,17,18], the prevalence of more recently described SE(-like) genes (*sey*, *selw*, *selx*, *selz*, *sel26*, and *sel27*) has not yet been well characterized.

*sey* was first described as a SET-like gene having 32% amino acid sequence identity to SET, and phylogenetically related to the group I superantigen including the TSST-1 gene [19]. This gene was identified in isolates from food poisoning, skin diseases, nasal colonization, and bovine mastitis, and its recombinant protein was proved to have superantigen activity in human mononuclear cells and emetic activity in a primate animal [19,20]. *selx*, which is classified as a group I superantigen, was revealed to be present at a high rate in the core genome of genetically diverse *S. aureus* strains [21,22]. In addition to superantigenic activity, SELX has an ability to bind to neutrophils, which inhibits its phagocytosis function, and thus is presumed to be implicated in the virulence of CA-MRSA [23]. *selz*, which belongs to SEB group (group II), was reported in the RF122 strain from bovine mastitis [4]. *sel26* and *sel27* (GenBank accession no. MF370874) were reported in *S. aureus*, *S. argenteus* and *S. schweizeri* and assigned into SEI and SEB groups (group V and II), respectively [5].

*selw* was reported as a novel SE-like gene by Okumura et al. [24] in the *S. aureus* strain N315 (GenBank accession no. BA000018, locus_tag SA1430) based on the nomenclature standard of SE [25]. It exhibited similarity to *sea* (36% amino acid sequence identity) and was classified into the same phylogenetic group as SEA (group III) [24]. *selw* had been previously used to refer to *selu2* [26,27,28,29], an allelic variant of *selu*; both *selu* variants are phylogenetically distinct from the gene described by Okumura et al. [4,30,31]. Thus, *selw* has been discriminated from *selu2* [4,8,31,32]. In the present study, *selw* denotes the SE-like gene that was described for the N315 strain [24].

We previously analyzed 624 CA-MRSA clinical isolates that were derived from outpatients in Hokkaido, Northern main island of Japan, for their molecular epidemiological and genetic characteristics [33], and reported the predominance of SCC*mec* IIa MRSA, and also the presence of SCC*mec* IVa-ST8 isolates (USA300 clone) carrying Panton–Valentine leukocidine (PVL) genes and ST5/ST764 MRSA-harboring arginine catabolic mobile element (ACME). Furthermore, we identified ST8 MRSA as having SCC*mec* IVl, which had been designated “CA-MRSA-J” and presumably emerged in Japan and other regions of Asia. In the present study, the prevalence of the newly described SE(-like) genes (*sey*, *selw*, *selx*, *selz*, *sel26*, and *sel27*) in these CA-MRSA isolates were investigated and their genetic diversity was analyzed phylogenetically.

## 2. Results

### 2.1. The Prevalence of sey, selw, selx, selz, sel26, and sel27

The prevalence of the SE(-like) genes among 624 CA-MRSA isolates is summarized in Table 1 and SE(-like) gene profiles in different sequence types (STs) of the selected 100 isolates are shown in Table 2. The most prevalent SE(-like) gene was *selw* (92.9%), followed by *selx* (85.6%), *sey* (35.4%), and *selz* (5.6%), while no isolates harbored *sel26* and *sel27*. *selw* was commonly detected in isolates with genotypes *coa*-IIa-ST5/ST764 (98.9%), *coa*-VIIa-ST1 (90.2%), and *coa*-IIIa-ST8 (86.5%), and also found in *coa*-Va-ST121, *coa*-Ia-ST89, and *coa*-VIIb-ST45 isolates. *spa* types t002, t1784, t008 were the most common in *coa*-IIa-ST5/764, *coa*-VIIa-ST1, and *coa*-IIIa-ST8, respectively. While *sey* showed a high prevalence in *coa*-IIa-ST5/764 and *coa*-Va-ST121, this gene was less frequently detected in *coa*-IIIa-ST8 and *coa*-VIIa-ST1 (30–40%). *selx* was prevalent in *coa*-Va-ST121, *coa*-VIIa-ST1, *coa*-IIa-ST5/764, and *coa*-IIIa-ST8 with a detection rate of more than 80%. *selz* was identified at a high rate in only *coa*-Va-ST121 and *coa*-Ia-ST89 isolates.

The profiles of SE(-like) genes were generally unique to the STs of isolates (Table 2). Panton–Valentine Leukocidin/arginine catabolic mobile element (PVL/ACME)-positive ST8 (SCC*mec*IVa-t008, USA300 clones) isolates had only four SE(-like) genes (*sek*, *seq*, *selw* and *selx*), while ST1, ST5, ST764 isolates harbored more genes with high rates of *selw* and *selx*. PVL/ACME-negative ST8 isolates of the CA-MRSA/J clone had *sec*, *sel*, and *sep,* in addition to *selw* and *selx*, while non-CA-MRSA/J ST8 isolates exhibited different profiles of SE(-like) genes.

Co-detection of *selw-selx-sey-selz* was found in ST121 and ST89 MRSA isolates, while *selw-selx-sey* was found in ST5 and its SLV (ST764 and ST5425), ST8 (PVL+/ACME+, non-CA-MRSA/J), and ST45 isolates. Though *sey*, *selw, selx*, and *selz* are not located in an enterotoxin gene cluster (*egc*) in the chromosome of *S. aureus* [28,30], *egc-2* (*seg-sei-sem-sen-seo-seu*) was co-detected with *sey*/*selw*/*selx*/*selz* in ST5 and ST764 (CC5), and also in ST121. *egc-1* (*seg-sei-sem-sen-seo)* was found in ST45 and ST5425 isolates, together with *selw* and *selx*.

*sey* and *selz* were more commonly identified in SCC*mec* V MRSA than in SCC*mec* II and III isolates. No distinct difference was found in the prevalence of *selw* and *selx* depending on SCC*mec* types. Detection rates of the SE(-like) genes analyzed in the present study were generally similar among the different specimens from which MRSA isolates were derived.

### 2.2. Phylogenetic and Sequence Analysis of sey, selw, selx, and selz

**Figure 1:**
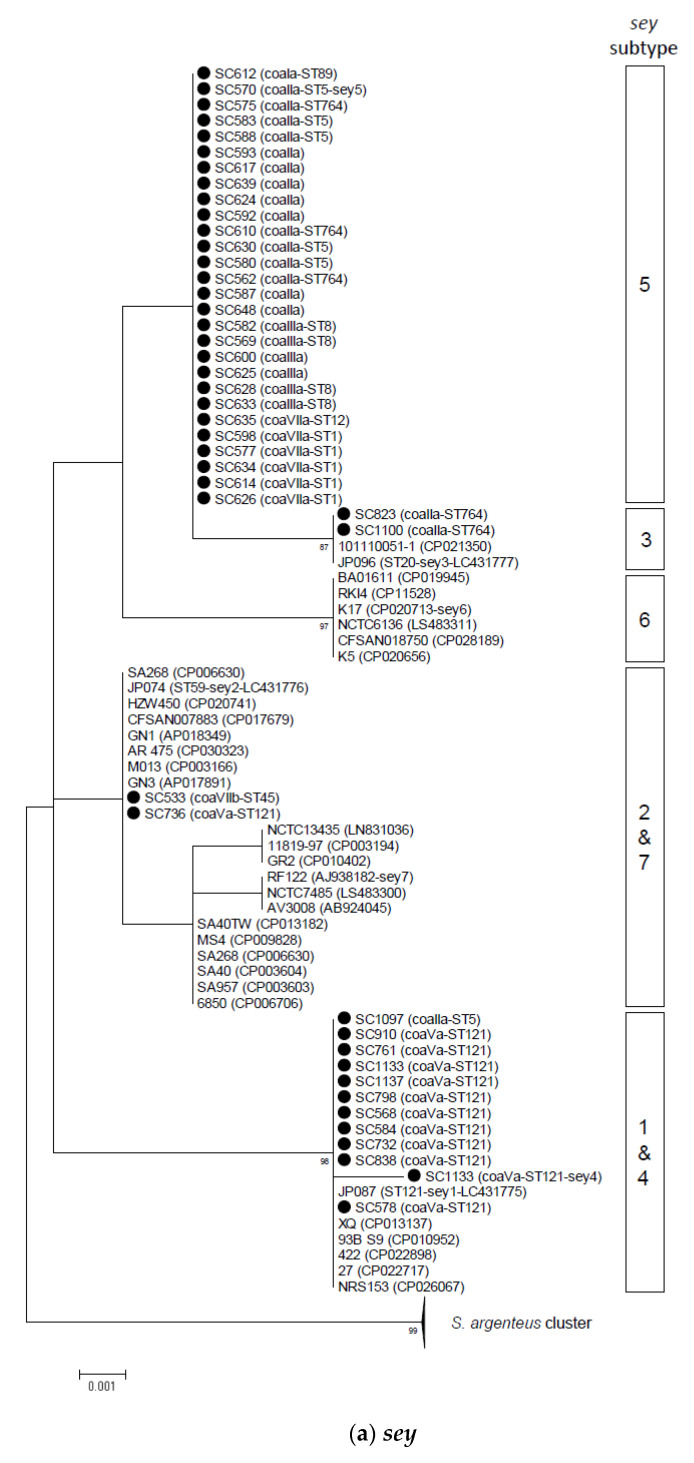

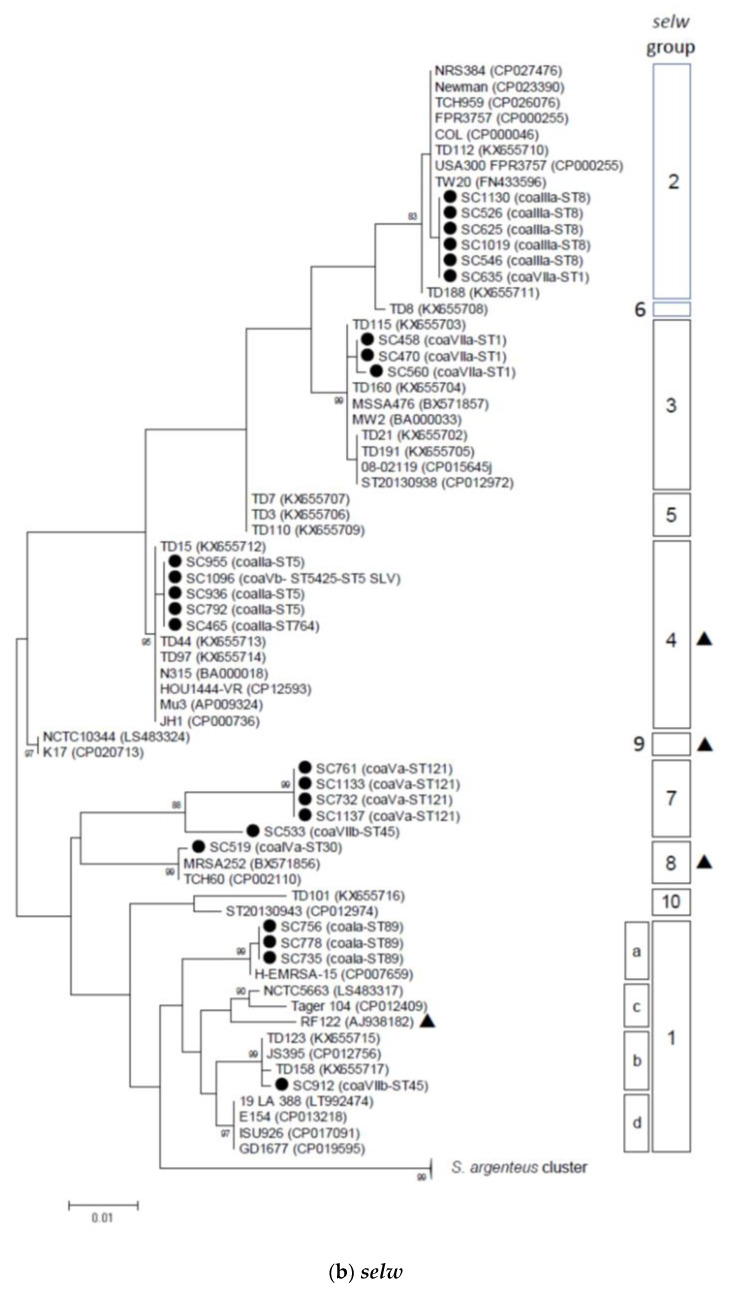

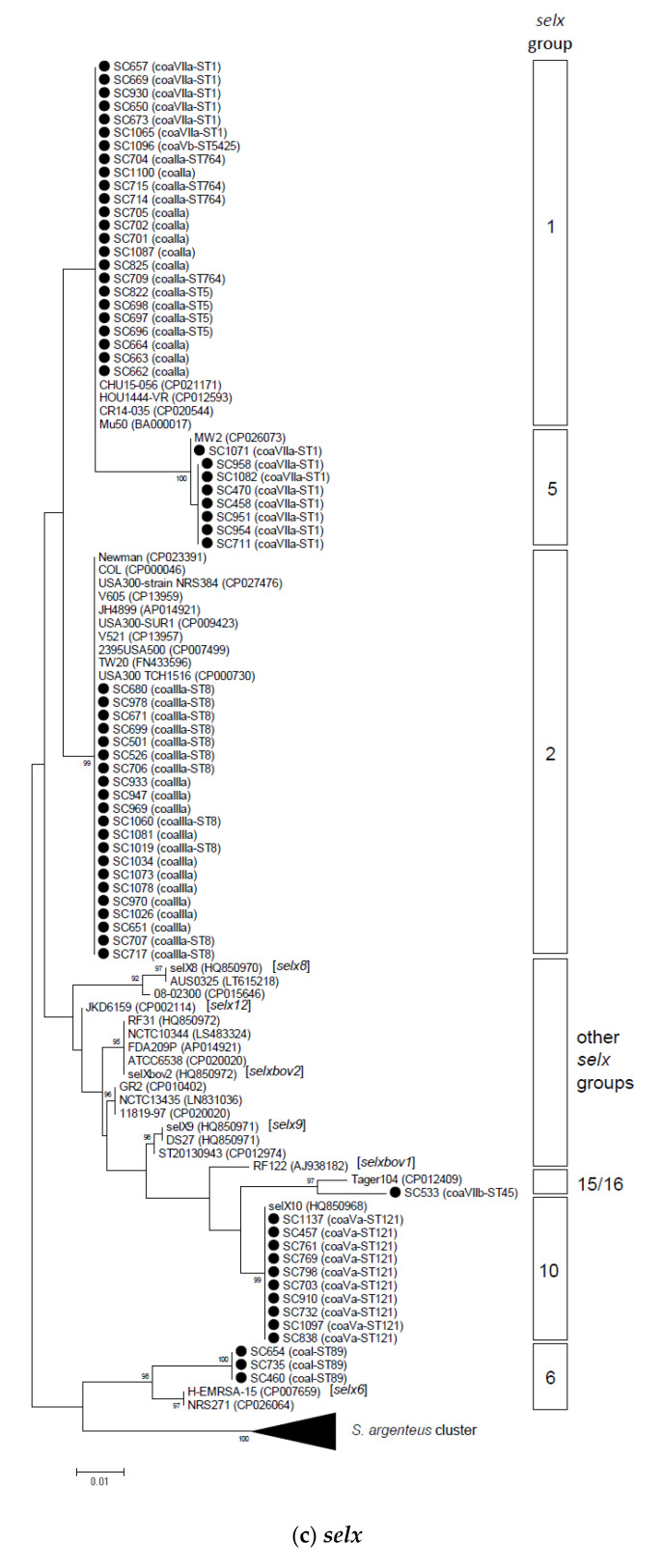

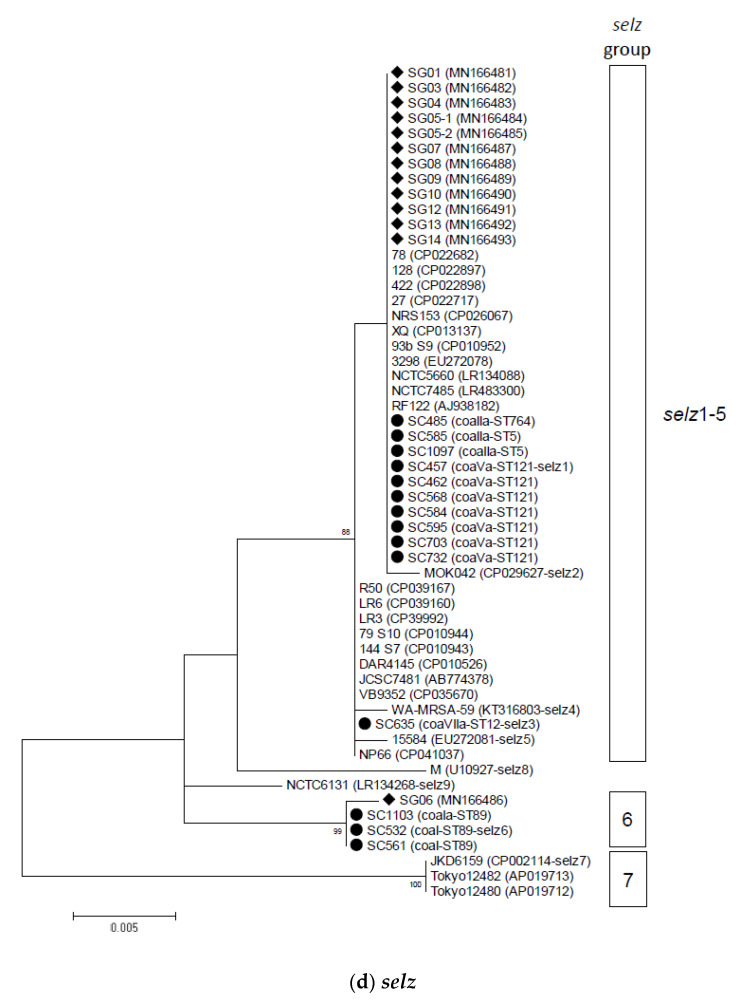
Phylogenetic dendrogram of *sey* (**a**), *selw* (**b**), *selx* (**c**), *selz* (**d**) constructed by the maximum likelihood method using MEGA6. The tree was statistically supported by bootstrapping with 1000 replicates, and genetic distances were calculated by the Kimura two-parameter model. The variation scale is provided at the bottom. The percentage bootstrap support is indicated by the values at each node (values <80 are omitted). The isolates analyzed in the present study are shown with closed circles. The *S. argenteus* cluster in *sey*, *selw*, and *selx* is shown at the bottom ((**a**), (**b**), (**c**)), while *selz* of the *S. argenteus* strain is indicated by a diamond (**d**). Subtypes/groups of individual SE(-like) genes are shown by boxes on the right. Closed triangles with *selw* groups and a strain in (**b**) represent genes encoding truncated proteins. Genotypes of isolates or GenBank accession numbers are shown in parenthesis followed by strain names.

For the 149 selected isolates belonging to different *coa* genotypes, nucleotide sequences of full-length ORF of *sey*, *selw*, *selx*, and *selz* were determined (44, 24, 67, and 14 isolates, respectively). Phylogenetic trees of these genes were constructed by the maximum likelihood method for the SE(-like) genes analyzed in the present study together with sequences in the GenBank database for representative *S. aureus* strains and those representing subtypes of individual SE(-like) genes (Figure 1).

SE gene *sey* was genetically differentiated into at least seven subtypes (*sey*1–*sey*7), including three variants, (*sey*1–*sey*3) described by Aziz et al. [20] (Figure 1a, Figure S1). The nucleotide sequence identity among the seven *sey* subtypes was more than 98.8% (Table S1). Phylogenetically, *sey*1 and *sey*4, and *sey*2 and *sey*7 were assigned into a same group and *sey*5 was genetically close to *sey*3, having only one nucleotide (amino acid) difference. *sey* sequences of the CA-MRSA isolates were mostly assigned into *sey*5, which included various genotypes, i.e., *coa*-Ia-ST89, *coa*-IIa-ST5/ST764, *coa*-IIIa/ST8, and *coa*-VIIa-ST1/ST12. The second most common subtype was *sey*1, which was identified in *coa*-Va-ST121 and *coa*-IIa-ST5 isolates.

*selw* had been classified into six groups (1–6) in our previous study on colonizing *S. aureus* isolates from food handlers [11]. In the present study, ten *selw* groups (group 1–10), including six groups previously reported, were discriminated, and group 1 was subdivided into four clusters (1a–1d) (Figure 1b, Figure S2). *selw* sequences were highly divergent, exhibiting >93% identity among all the groups, and 95–98% within group 1 (Table S2). Truncated products deduced from *selw* sequences were identified in isolates of groups 4, 8, and 9, and RF122 strain (group 1c). These were caused by internal stop codons, resulting in a lack of 85–130 amino acids at the C-terminal portion, while intact SElW consists of 250 amino acids (Figure S2). *selw* of the CA-MRSA isolates was assigned into groups 1 (1a,b), 2, 3, 4, 7, and 8, which contained isolates with *coa*-Ia-ST89/*coa*-VIIb-ST45, *coa*-IIIa/VIIa-ST1/ST8, *coa*-VIIa-ST1, *coa*-IIa-ST5 (CC5), *coa*-Va-ST121, and *coa*-IVa-ST30, respectively.

Wilson et al. classified *selx* into at least 14 alleles (subtypes) [21] having a sequence identity of >94% (Table S3). According to this classification, most of the *selx* in the CA-MRSA isolates were assigned into *selx*1, *selx*2, *selx*5, and *selx*10, which contained *coa*-IIa/VIIa-ST1/ST5/ST764, *coa*-IIIa-ST8, *coa*-VIIa-ST1, and *coa*-Va-ST121 isolates, respectively (Figure 1c, Figure S3). Only isolate SC533 was assigned into a new subtype, *selx*16. Alignment of SElX amino acid sequences revealed that a sialic acid-binding region consisting of 16 amino acids [22,23] is conserved among all of the subtypes, except for a single position (Figure S3).

*selz* sequences determined in the present study and those obtained from the GenBank database were classified into nine subtypes (*selz*1–*selz*9), among which *selz*1–*selz*5 were phylogenetically assigned to a single group (*selz*1 group) (Figure 1d, Figure S4). Nucleotide sequence identity among different *selz* subtypes was 96–99% (Table S4). *selz* of the CA-MRSA was assigned to *selz*1 and *selz*6 groups, which were identified in *coa*-Va-ST121/*coa*-IIa-ST5/764 and *coa*-Ia-ST89 isolates, respectively. *selz* of *S. argenteus* and *S. aureus* were classified into the same group (*selz*1 and *selz*6 groups), although *S. argenteus* clusters of *sey*, *selw*, and *selx* were distinct from that of *S. aureus* (Figure 1a–d).

## 3. Discussion

In this study, we investigated the prevalence of six SE(-like) genes (*sey*, *selw*, *selx*, *selz*, *sel26*, and *sel27*) in CA-MRSA clinical isolates and revealed a high prevalence of *selw* and *selx*, a lower prevalence of *sey* and *selz*, and an absence of *sel26* and *sel27*. It was notable that these six genes are distributed also to *S. argenteus*, although the prevalence of other SE(-like) genes was very low, except for *sec* and the enterotoxin gene cluster (*egc*, *seg-sei-sem-sen-seo*) in some isolates [34]. The prevalence of SE(-like) genes analyzed in the present study was different among *S. argenteus* depending on lineages: *sey*, *sel26* and *sel27* in ST2250; *selx* in ST2198, and *selw* in ST1223, while *selz* was found in all three STs [34]. Phylogenetically, *sey*, *selw*, and *selx* of *S. argenteus* formed a distinct cluster from those of *S. aureus*. In contrast, *selz* was not genetically differentiated by the two staphylococcal species. These findings suggest that *sey*, *selw*, *selx*, and *selz* might have been archaic virulence determinants in *S. aureus* complex (SAC) and passed on to progeny through their deletion and genetic evolution in individual lineages of *S. aureus* and *S. argenteus*. Although the presence of *sel26* and *sel27* was reported in ST27 and ST772 *S. aureus* strains in addition to ST2250 *S. argenteus* strains [5], our present study suggests that these genes may not play an important role in the virulence of CA-MRSA.

A high prevalence of *selx* was reported for *S. aureus* clinical isolates from blood, diabetic foot ulcers, and cystic fibrosis, as well as for those from colonization [8,9,10,11], showing comparable or higher detection rates than those of *sea*, *sec*, and *egc*. Furthermore, *selx* was revealed to be highly conserved, despite the presence of various subtypes. Therefore, it is suggested that *selx* may be involved in any universal role in the virulence of *S. aureus*, which is presumably associated with superantigenic activity as well as neutrophil-binding activity [21,23]. Though SELlX was initially reported as a novel virulence factor for the USA300 clone [21], this SE-like protein may be implicated in pathogenesis of broad CA-MRSA clones. Although *selx* was differentiated into more than 16 subtypes, including newly assigned types in the present study, all the SElX subtypes have a conserved motif of sialic acid binding [22,23], which is essential for its superantigen- and neutrophil-binding activity. Thus, the biological function of SElX is suggested to be same among its subtypes.

In contrast, *selw* was revealed to be divergent and classified into ten genetic groups. Four *selw* groups contained putative pseudogenes that encode truncated products, which lose 30–50% amino acids of intact SElW. Thus, such truncated products are suggested to be dysfunctional or have reduced function compared with intact SElW. The highly divergent nature of *selw* may suggest that this gene is a remnant of archaic, universal virulence determinant in SAC, having a less significant role in virulence than *selx*.

SEY, with three subtypes (*sey*1–*sey*3), was reported as a novel SE as well as superantigen in *S. aureus* and identified in isolates from skin diseases with a detection rate of 17–22% [20]. In our present study for CA-MRSA, a higher prevalence (35.4%) was noted, particularly in *coa*-I-III, V and VII. In addition to the three subtypes described previously, a new subtype *sey*5, which is genetically related to *sey*3, was identified and contained in a higher number of isolates belonging to various genotypes (*coa*-Ia/IIa/IIIa/VIIa-ST1/5/8/12/89/764) than *sey*1 (*coa*-IIa/Va-ST5/121). Thus, the *sey* variant group comprising *sey*5 and *sey*3 may be more prevalent among CA-MRSA, presumably associated with its virulence. Aziz et al. [20] reported that the effects of SEY on human lymphocytes were slightly different among SEY variants (SEY1, SEY2, SEY3), but not significantly. Because the additional SEY subtypes (SEY4-7) detected in our study were genetically close to all of SEY1-3 and the divergent amino acid positions were also similar, the newly identified SEY subtypes probably have the same function as that reported for SEY1-3.

It was notable in this study that ST121 isolates (*coa*-Va) had *sey*, *selw*, *selx*, and *selz*, leading to the highest detection rates of *sey* and *selz* in *coa*-Va. Because the ST121 *S. aureus* clone is characterized by various virulence factors and referred to as “hypervirulent” [35], *sey*, *selw*, *selx*, and *selz* are also suggested to be involved in increased virulence in this clone. The putative ubiquity of *selw* and *selx* and the prevalence of *sey* and *selz* in some specific lineages (e.g., ST121) in CA-MRSA may provide the possibility for their potential pathogenic role in human infections.

CA-MRSA has been reported to possess mostly SCC*mec* IV or V in clones represented by ST1, ST8, ST30, ST59, and ST80 [2]. However, in our previous studies in northern Japan, SCC*mec* II was predominant among CA-MRSA, accounting for more than 72%, while remaining MRSA had SCC*mec* IV or V [33,36]. SCC*mec* II-MRSA with ST5, which is known as the “New York/Japan clone, has been one of the typical HA-MRSA clones and detected predominantly in our previous studies [37,38]. Thus, our present results may represent the situation of the SE(-like) gene in an area where SCC*mec* II is dominant among CA-MRSA. Further study may be necessary to reveal the prevalence of the newer SE genes among CA-MRSA with type IV and V SCC*mec*.

This study revealed the ubiquitous distribution of *selw* and *selx* in CA-MRSA and the prevalence of *sey* and *selz* dependent on some specific lineages. These findings suggest a potential role of these newly described SE(-like) in the pathogenicity of CA-MRSA.

## 4. Materials and Methods

### 4.1. CA-MRSA Isolates

A total of 624 non-duplicate CA-MRSA isolates, which had been analyzed for their molecular epidemiological characteristics (SCC*mec*, ST, *coa*-type, etc.) in our previous study [33], were used as the subject of the present study. These isolates were derived from clinical specimens of outpatients who visited hospitals and clinics in the prefecture of Hokkaido, Japan, during a nine-month period (July 2013 and March 2014). Coagulase genotype (*coa*) is the genetic classification of the staphylocoagulase gene based on its diversity in N-terminal divergent regions (D1, D2) and is discriminated by the multilex PCR scheme [39]. The most prevalent coagulase (*coa*) genotype was IIa (455, 72.9%), followed by IIIa (74, 11.9%), VIIa (51, 8.2%), Ia (17, 2.7%), and Va (16, 2.6%). The dominant SCCmec type was IIa (439, 70.4%) followed by IVa (78, 12.5%), V (34, 5.4%) IVl (24, 3.8%), and IVh (13, 2.1%) [33].

### 4.2. Genetic Analysis of S. aureus (CA-MRSA) Isolates

SE(-like) genes *sey*, *selw*, *selx*, *selz*, *sel26*, and *sel27* were detected by uniplex PCR, using primers listed in Table S5 The nucleotide sequences of the full-length ORF of SE(-like) genes were determined by direct sequencing with PCR products, using the BigDye Terminator v3.1 Cycle Sequencing Kit (Applied Biosystems, Foster City, CA, USA) on an automated DNA sequencer (ABI PRISM 3100). Primers were designed to amplify ORF of the SE(-like) genes in this study (Table S5). The phylogenetic dendrogram of toxin genes was constructed by the maximum likelihood method using the MEGA.6 software package. Multiple alignment of nucleotide/amino acid sequences determined in the present study and those retrieved from the GenBank database was performed by Clustal Omega program (https://www.ebi.ac.uk/Tools/msa/clustalo/), which was also used for the calculation of the sequence identity. In our previous study [33], the *coa* genotype and SCC*mec* type were determined for all the isolates, and *spa*, ST, and enterotoxin profiles were characterized for 31 isolates. In the present study, *spa* type and ST were determined for more representative 118 and 69 isolates, respectively, as described previously [33]. For the 69 isolates, of which ST was determined, the prevalence of all the SE(-like) genes was analyzed [38,40].

### 4.3. GenBank Accession Numbers

The nucleotide sequences of *sey*, *selw*, *selx*, *selz* were deposited in the GenBank database under the accession numbers listed in Table S6.

## Figures and Tables

**Table 1:** The prevalence of *sey*, *selw*, *selx* and *selz* among 624 CA-MRSA isolates with different genotypes, SCC*mec* types and origins.

Genotype		Total No. of Isolates	No. of Isolates With SE(-Like) Gene *^1^ (%)			
*coa* Genotype	*spa* Type (n = 149) *^2^		*sey*	*selw*	*selx*	*selz*
Ia	t375 (3)	17	8 (47.1)	8 (47.1)	13 (76.5)	12 (70.6)
IIa	t002 (56), t548 (2), t2487 (2), t001 (1), t045 (2)	455	157 (34.5)	450 (98.9)	397 (87.3)	5 (1.1)
IIIa	t008 (9), t4133 (2), t1767 (24), t5071 (1), t1627 (2), t1581(2)	74	22 (29.7)	64 (86.5)	60 (81.1)	0
IVa	t019 (1)	3	0	1 (33.3)	0	0
Va	t5110 (6), t10641 (10)	16	13 (81.3)	8 (50)	16 (100)	14 (87.5)
Vb	NT (1)	1	0	1 (100)	1 (100)	0
VIa	ND	4	0	0	0	0
VIIa	t1784 (23)	51	20 (39.2)	46 (90.2)	46 (90.2)	4 (7.8)
VIIb	t370 (2)	3	1 (33.3)	2 (66.7)	1 (33.3)	0
Total		624	221 (35.4)	580 (92.9)	534 (85.6)	35 (5.6)
SCC*mec* type						
SCC*mec* I		2	0	1 (50)	2 (100)	0
SCC*mec* II		452	154 (34.1)	431 (95.4)	393 (86.9)	5 (1.1)
SCC*mec* IV		125	41 (32.8)	115 (92)	106 (84.8)	4 (3.2)
SCC*mec* V		34	26 (76.5)	30 (88.2)	30 (88.2)	26 (76.5)
SCC*mec* NT		11	0	3 (27.3)	3 (27.3)	0
Specimen						
sputum		136	45 (33.1)	130 (95.6)	121 (89)	9 (6.6)
urine		129	40 (31)	118 (91.5)	102 (79.1)	3 (2.3)
ear discharge		76	35 (46.1)	72 (94.7)	70 (92.1)	6 (7.9)
nasal discharge		75	28 (37.3)	73 (97.3)	67 (89.3)	7 (9.3)
pus		57	24 (42.1)	54 (94.7)	52 (91.2)	3 (5.3)
wound swab		29	13 (44.8)	27 (93.1)	27 (93.1)	3 (10.3)
eye swab		29	12 (41.4)	28 (96.6)	26 (89.7)	2 (6.9)
stool		33	9 (27.3)	26 (78.8)	24 (72.7)	0
skin		26	10 (38.5)	24 (92.3)	23 (88.5)	2 (7.7)
Others *^3^		34	5 (14.7)	28 (82.4)	22 (64.7)	0

NT, non-typable. *^1^ *sel26* and *sel27* were negative for all the isolates. *^2^ *spa* type and ST were determined for a total of 149 isolates comprising *coa*-Ia (3), *coa*-IIa (63), *coa*-IIIa (40), *coa*-IVa (1), *coa*-Va (16), *coa*-Vb (1) *coa*-VIIa (23), and *coa*-VIIb (2). ND, *spa*-typing not done. *^3^ Others included specimens of blood, bronchial lavage fluid, pharynx, aspirate, pleural fluid, joint fluid, HVS, catheter tip, drainage fluid, suction tube.

**Table 2:** The presence of enterotoxin(-like) genes in CA-MRSA isolates with different STs.

PVL/ACME Genes	ST (CC)	Total No. of Isolates (n = 100)	SE(-Like) Genes Identified *^2^
PVL+/ACME+	ST8 (CC8)	9 *^1^	*sek, seq, selw, selx*
PVL+/ACME-	ST30 (CC30)	1 *^1^	*sem, sen, seo, seu, selw, selx*
	ST59 (CC59)	1 *^1^	*seb, sek, seq, selw, selx*
PVL-/ACME+	ST5/ST764 (CC5)	15 *^1^	*seb* (67%), *sec* (20%), *seg, sei, sem, sen, seo, seu*, *sep* (33%), *selw*, *selx, sey*
PVL-/ACME-	ST8 (CC8) (CA-MRSA/J *^3^)	5 *^1^	*sec, sel, sep, selw, selx*
	ST8 (CC8)	14	*selj* (29%)*, ser* (29%*), selw* (93%)*, selx* (93%)*, sey* (43%)
	ST5/ST764 (CC5)	20	*seb* (60%), *sec* (10%), *seg, sei, sem, sen, seo, seu*, *selw*, *selx, sey* (30%)*, selz* (25%)
	ST5425 (CC5)	1	*seg, sei, sem, sen, seo, selw, selx*
	ST45 (CC45)	2	*seg, sei, sem, sen, seo, selw*, *selx, sey*
	ST1 (CC1)	13	*sea, sek, seq, selx* (92%)*, selw* (92%), *sey* (31%)*, selz* (15%)
	ST89 (CC89)	8	*sem, seo, seu, selw, selx, sey, selz*
	ST121 (CC121)	10	*seg, sei, sem, sen, seo, seu, selw* (50%), *selx, sey* (80%)*, selz* (85%)
	ST12 (CC12)	1	*sep, selw, selx, selz*

ST, sequence type; CC, clonal complex. *^1^ For these 31 isolates, genotypes and enterotoxin genes profile had been already reported in our previous study [33]. *^2^ None of isolate had *sed*, *see*, *ses* and *set*. When SE(-like) genes were not present in all the isolates of the same ST, their detection rate (%) are indicated in parentheses. ^*3^ CA-MRSA/J represents ST8 MRSA carrying SCC*mec* IVl.

## Supplementary Materials

The following are available online at https://www.mdpi.com/2072-6651/12/5/347/s1, Figure S1: Alignment of amino acid and nucleotide sequences of SEY, Figure S2: Alignment of SElW amino acid sequences of all the MRSA isolates analyzed in the present study, and representative strains of all the *selw* phylogenetic groups, Figure S3: Alignment of SElX amino acid sequences representing subtypes SElX1-SElX16, Figure S4: Alignment of SElZ amino acid sequences representing subtypes SElZ1-SElZ9, Tables S1–S4: Nucleotide Sequence identities (percentage) of *sey, selw*, *selx*, and *selz* gene among selected *S. arueus* isolates and those of reported *S. argenteus* strain, respectively, Table S5: Primers used in the present study, Table S6: GenBank accession numbers assigned to *sey*, *selx*, *selw* and *selz* sequences determined in the present study.
