# Supplementary material for: Prevalence and Genetic Diversity of Staphylococcal Enterotoxin (-Like) Genes sey, selw, selx, selz, sel26 and sel27 in Community-Acquired Methicillin-Resistant Staphylococcus aureus
Source: Toxins (Basel). 2020 May 23;12(5):347. doi: 10.3390/toxins12050347 (PMC7291287; doi:10.3390/toxins12050347)
Supplement: Supplementary file 1 [file toxins-12-00347-s001.pdf]

Supplementary Materials: Prevalence and Genetic Diversity of Staphylococcal Enterotoxin (-like) Genes *sey*, *selw*, *selx*, *selz*, *sel26* and *sel27* in Community-Acquired Methicillin-Resistant *Staphylococcus aureus*

Meiji Soe Aung, Noriko Urushibara, Mitsuyo Kawaguchiya, Masahiko Ito, Satoshi Habadera and Nobumichi Kobayashi

(a)

```
JP087-sey1      MKAKLCFLLTTLAFLIAVTGSIGIAEVKAKTTGLITENSNDSLKEHYAQKFEVYTNKEVT    60
SC1133-sey4     MKAKLCFLLTTLAFLIAVTGSIGIAEVKAKTTGLITENSNDSLKEHYAQKFEVYTNKEVT    60
JP096-sey3      MKAKLWFLLTTLAFLIAVTGSIGIAEVKAKTTGLITENSNDSLKEHYAQKFEVYTNKEIT    60
SC570-sey5      MKAKLWFLLTTLAFLIAVTGSIGIAEVKAKTTGLITENSNDSLKEHYAQKFEVYTNKEVT    60
RF122-sey7      MKAKLWFLLTTLAFLIAVTESIGIAEVKAKTTGLITENSNDSLKEHYAQKFEVYTNKEVT    60
JP074-sey2      MKAKLWFLLTTLAFLIAVTGSIGIAEVKAKTTGLITENSNDSLKEHYAQKFEVYTNKEVT    60
RK14-sey6       MKAKLWFLLTTLAFLIAVTGSIGIAEVKAKTTGLITENSNDSLKEHYAQKFEVYTNKEVT    60
                ***** ************* **************************************:*

JP087-sey1      GVGENYIDAKVDTYNVRTVLYNTDYLKQFKNQDKVNIWGTLYENQQSKVYRGTVVKYDPI    120
SC1133-sey4     GVGENYIDAKVDTYNVRTVLYNTDYLKQFKNQDKVNIWGTLYENQQSKVYRGTVVKYDPI    120
JP096-sey3      GVGENYIDAKVDTYNVRTVLYNTDYLKQFKNQDKVNIWGTLYENKQSKVYRGTVVKYDPI    120
SC570-sey5      GVGENYIDAKVDTYNVRTVLYNTDYLKQFKNQDKVNIWGTLYENKQSKVYRGTVVKYDPI    120
RF122-sey7      GVGENYIDTKVDTYNVRTVLYNTDYLKQFKNQDKVNIWGTLYENQQSKVYRGTVVKYDPI    120
JP074-sey2      GVGENYIDTKVDTYNVRTVLYNTDYLKQFKNQDKVNIWGTLYENQQSKVYRGTVVKYDPI    120
RK14-sey6       GVGENYIDVKVDTYNVRTVLYNTDYLKQFKNQDKVNIWGTLYENQQSKVYRGTVVKYDPI    120
                ********.************************************:**************

JP087-sey1      SKVTNLSYRMNLFVNGHQTKVNPDSLLEVKNKQISLKETDFRIRKYLLEKEHLYSNYNSG    180
SC1133-sey4     SKVTNLSYRMNLFVNGHQTKVNPDSLLEVKNKQISLKETDFRIRKYLLEKEHLYSNYNSG    180
JP096-sey3      SKVTNLSYRMNLFVNGHQTKVNPDSLLEVKNKQISLKETDFRIRKYLLEKEHLYSNYNSG    180
SC570-sey5      SKVTNLSYRMNLFVNGHQTKVNPDSLLEVKNKQISLKETDFRIRKYLLEKEHLYSNYNSG    180
RF122-sey7      SKVTNLSYRMNLFVNGHQTKVNPDSLLEVKNKQISLKETDFRIRKYLLEKEHLYSNYNSG    180
JP074-sey2      SKVTNLSYRMNLFVNGHQTKVNPDSLLEVKNKQISLKETDFRIRKYLLEKEHLYSNYNSG    180
RK14-sey6       SKVTNLSYRMNLFVNGHQTKVNPDSLLEVKNKQISLKETDFRIRKYLLEKEHLYSNYNSG    180
                ************************************************************

JP087-sey1      ELIVEMKNGARHKIDLGDILSDSQEKTFDFDNISHIDIYMK      221
SC1133-sey4     ELIVEMKNGARHKIDLCDILSDSQEKTFDFDNISHIDIYMK      221
JP096-sey3      ELIIEMKNGARHKIDLGDILSDSQEKTFDFDNISHIDIYMK      221
SC570-sey5      ELIIEMKNGARHKIDLGDILSDSQEKTFDFDNISHIDIYMK      221
RF122-sey7      ELIIEMKNGARHKIDLGDILSDSQEKTFDFDNISHIDIYMK      221
JP074-sey2      ELIIEMKNGARHKIDLGDILSDSQEKTFDFDNISHIDIYMK      221
RK14-sey6       ELIIEMKNGARHKIDLGDILSDSQEKTFDFDNISHIDIYMK      221
                ***:************ ************************
```

(b)

```
sey4    ATGAAAGCGAAACTATGTTTTTTATTGACAACTTTGGCATTTTTGATTGCAGTAACGGGA    60
sey1    ATGAAAGCGAAACTATGTTTTTTATTGACAACTTTGGCATTTTTGATTGCAGTAACGGGA    60
sey6    ATGAAAGCTAAATTATGGTTTTTATTGACAACTTTGGCATTTTTGATTGCAGTAACGGGA    60
sey2    ATGAAAGCGAAACTATGGTTTTTATTGACAACTTTGGCATTTTTGATTGCAGTAACGGGA    60
sey7    ATGAAAGCGAAACTATGGTTTTTATTGACAACTTTGGCATTTTTGATTGCAGTAACGGAA    60
sey5    ATGAAAGCTAAACTATGGTTTTTATTGACAACTTTGGCATTTTTGATTGCAGTAACGGGA    60
sey3    ATGAAAGCTAAACTATGGTTTTTATTGACAACTTTGGCATTTTTGATTGCAGTAACGGGA    60
        ******** *** **** **************************************** *
```

```
sey4        TCTATTGGAATAGCAGAAGTAAAAGCAAAAACAACTGGATTGATTACAGAAAATAGTAAT    120
sey1        TCTATTGGAATAGCAGAAGTAAAAGCAAAAACAACTGGATTGATTACAGAAAATAGTAAT    120
sey6        TCTATTGGAATAGCAGAAGTAAAAGCAAAAACAACTGGATTGATTACAGAAAATAGTAAT    120
sey2        TCTATTGGAATAGCAGAAGTAAAAGCAAAAACAACTGGATTGATTACAGAAAATAGTAAT    120
sey7        TCTATTGGAATAGCAGAAGTAAAAGCAAAAACAACTGGATTGATTACAGAAAATAGTAAT    120
sey5        TCTATTGGAATAGCAGAAGTAAAAGCAAAAACAACTGGATTGATTACAGAAAATAGTAAT    120
sey3        TCTATTGGAATAGCAGAAGTAAAAGCAAAAACAACTGGATTGATTACAGAAAATAGTAAT    120
            ************************************************************

sey4        GACAGTTTAAAAGAGCATTATGCACAAAAATTTGAAGTTTATACGAATAAAGAAGTAACA    180
sey1        GACAGTTTAAAAGAGCATTATGCACAAAAATTTGAAGTTTATACGAATAAAGAAGTAACA    180
sey6        GACAGTTTAAAAGAGCATTATGCACAAAAATTTGAAGTTTATACGAATAAAGAAGTAACA    180
sey2        GACAGTTTAAAAGAGCATTATGCACAAAAATTTGAAGTTTATACGAATAAAGAAGTAACA    180
sey7        GACAGTTTAAAAGAGCATTATGCACAAAAATTTGAAGTTTATACGAATAAAGAAGTAACA    180
sey5        GACAGTTTAAAAGAGCATTATGCACAAAAATTTGAAGTTTATACGAATAAAGAAGTAACA    180
sey3        GACAGTTTAAAAGAGCATTATGCACAAAAATTTGAAGTTTATACGAATAAAGAAATAACA    180
            ****************************************************** *****

sey4        GGAGTTGGGGAAAATTATATAGACGCGAAAGTTGACACCTACAATGTACGGACAGTGCTC    240
sey1        GGAGTTGGGGAAAATTATATAGACGCGAAAGTTGACACCTACAATGTACGGACAGTGCTC    240
sey6        GGAGTTGGGGAAAATTATATAGACGTGAAAGTTGACACCTACAATGTACGGACAGTGCTC    240
sey2        GGAGTTGGGGAAAATTATATAGACACGAAAGTTGACACCTACAATGTACGGACAGTGCTC    240
sey7        GGAGTTGGGGAGAATTATATAGACACGAAAGTTGACACCTACAATGTACGGACAGTGCTC    240
sey5        GGAGTTGGGGAAAATTATATAGACGCGAAAGTTGACACCTACAATGTACGGACAGTGCTC    240
sey3        GGAGTTGGGGAAAATTATATAGACGCGAAAGTTGACACCTACAATGTACGGACAGTGCTC    240
            *********** ************  **********************************

sey4        TACAACACTGATTATTTGAAACAGTTTAAAAATCAAGACAAAGTTAATATATGGGGAACA    300
sey1        TACAACACTGATTATTTGAAACAGTTTAAAAATCAAGACAAAGTTAATATATGGGGAACA    300
sey6        TACAACACTGATTATTTGAAACAGTTTAAAAATCAAGACAAAGTTAATATATGGGGAACA    300
sey2        TACAACACTGATTATTTGAAACAGTTTAAAAATCAAGACAAAGTTAATATATGGGGAACA    300
sey7        TACAACACTGATTATTTGAAACAGTTTAAAAATCAAGACAAAGTTAATATATGGGGAACA    300
sey5        TACAACACTGATTATTTGAAACAGTTTAAAAATCAAGACAAAGTTAATATATGGGGAACA    300
sey3        TACAACACTGATTATTTGAAACAGTTTAAAAATCAAGACAAAGTTAATATATGGGGAACA    300
            ************************************************************

sey4        TTATATGAAAACCAACAATCTAAAGTATATCGTGGCACAGTAGTTAAGTATGATCCTATA    360
sey1        TTATATGAAAACCAACAATCTAAAGTATATCGTGGCACAGTAGTTAAGTATGATCCTATA    360
sey6        TTATATGAAAACCAACAATCTAAAGTATATCGTGGTACAGTAGTTAAGTATGATCCTATA    360
sey2        TTATATGAAAACCAACAATCTAAAGTATATCGTGGCACAGTAGTTAAGTATGATCCTATA    360
sey7        TTATATGAAAACCAACAATCTAAAGTATATCGTGGCACAGTAGTTAAGTATGATCCTATA    360
sey5        TTATATGAAAACAAACAATCTAAAGTATATCGTGGCACAGTAGTTAAGTATGATCCTATA    360
sey3        TTATATGAAAACAAACAATCTAAAGTATATCGTGGCACAGTAGTTAAGTATGATCCTATA    360
            ************ ********************** ************************

sey4        TCAAAAGTAACTAACTTATCTTATAGAATGAACTTGTTTGTTAACGGTCATCAAACTAAA    420
sey1        TCAAAAGTAACTAACTTATCTTATAGAATGAACTTGTTTGTTAACGGTCATCAAACTAAA    420
sey6        TCAAAAGTAACTAACTTATCTTATAGAATGAACTTGTTTGTTAACGGTCATCAAACTAAA    420
sey2        TCAAAAGTAACTAACTTATCTTATAGAATGAACTTGTTTGTTAACGGTCATCAAACTAAA    420
sey7        TCAAAAGTAACTAACTTATCTTATAGAATGAACTTGTTTGTTAACGGTCATCAAACTAAA    420
sey5        TCAAAAGTAACTAACTTATCTTATAGAATGAACTTGTTTGTTAACGGTCATCAAACTAAA    420
sey3        TCAAAAGTAACTAACTTATCTTATAGAATGAACTTGTTTGTTAACGGTCATCAAACTAAA    420
            ************************************************************

sey4        GTCAATCCAGACAGTTTATTAGAAGTTAAGAATAAACAGATTTCTCTAAAAGAAACAGAT    480
sey1        GTCAATCCAGACAGTTTATTAGAAGTTAAGAATAAACAGATTTCTCTAAAAGAAACAGAT    480
sey6        GTGAATCCAGACAGTTTATTAGAAGTTAAGAATAAACAGATTTCTCTAAAAGAAACAGAT    480
sey2        GTGAATCCAGACAGTTTATTAGAAGTTAAGAATAAACAGATTTCTCTAAAAGAAACAGAT    480
sey7        GTGAATCCAGACAGTTTATTAGAAGTTAAGAATAAACAGATTTCTCTAAAAGAAACAGAT    480
sey5        GTGAATCCAGACAGTTTATTAGAAGTTAAGAATAAACAGATTTCTCTAAAAGAAACAGAT    480
sey3        GTGAATCCAGACAGTTTATTAGAAGTTAAGAATAAACAGATTTCTCTAAAAGAAACAGAT    480
            ** *********************************************************
```

```
sey4       TTTAGAATTAGAAAATATTTATTAGAAAAAGAACACCTATATAGTAATTACAATTCAGGG    540
sey1       TTTAGAATTAGAAAATATTTATTAGAAAAAGAACACCTATATAGTAATTACAATTCAGGG    540
sey6       TTTAGAATTAGAAAATATTTATTAGAAAAAGAACACCTATATAGTAATTACAATTCAGGG    540
sey2       TTTAGAATTAGAAAATATTTATTAGAAAAAGAACACCTATATAGTAATTACAATTCAGGG    540
sey7       TTTAGAATTAGAAAATATTTATTAGAAAAAGAACACCTATATAGTAATTACAATTCAGGG    540
sey5       TTTAGAATTAGAAAATATTTATTAGAAAAAGAACACCTATATAGTAATTACAATTCAGGG    540
sey3       TTTAGAATTAGAAAATATTTATTAGAAAAAGAACACCTATATAGTAATTACAATTCAGGT    540
           ***********************************************************

sey4       GAACTAATTGTAGAAATGAAAAATGGAGCAAGACATAAAATAGATTTATGCGACATATTG    600
sey1       GAACTAATTGTAGAAATGAAAAATGGAGCAAGACATAAAATAGATTTAGGCGACATATTG    600
sey6       GAACTAATTATAGAAATGAAAAATGGAGCAAGACATAAAATAGATTTAGGCGACATATTG    600
sey2       GAACTAATTATAGAAATGAAAAATGGAGCAAGACATAAAATAGATTTAGGCGACATATTG    600
sey7       GAACTAATTATAGAAATGAAAAATGGAGCAAGACATAAAATAGATTTAGGCGACATATTG    600
sey5       GAACTAATTATAGAAATGAAAAATGGAGCAAGACATAAAATAGATTTAGGCGACATATTG    600
sey3       GAACTAATTATAGAAATGAAAAATGGAGCAAGACATAAAATAGATTTAGGCGACATATTG    600
           ********* ************************************** ***********

sey4       AGCGACTCACAAGAAAAAAACGTTTGATTTTGATAATATTAGTCATATAGATATTTATATG    660
sey1       AGCGACTCACAAGAAAAAAACGTTTGATTTTGATAATATTAGTCATATAGATATTTATATG    660
sey6       AGTGACTCACAAGAAAAAAACGTTTGATTTTGATAATATTAGTCATATAGATATTTATATG    660
sey2       AGTGACTCACAAGAAAAAAACGTTTGATTTTGATAATATTAGTCATATAGATATTTATATG    660
sey7       AGTGACTCACAAGAAAAAAACGTTTGATTTTGATAATATTAGTCATATAGATATTTATATG    660
sey5       AGTGACTCACAAGAAAAAAACGTTTGATTTTGATAATATTAGTCATATAGATATTTATATG    660
sey3       AGTGACTCACAAGAAAAAAACGTTTGATTTTGATAATATTAGTCATATAGATATTTATATG    660
           ** *********************************************************

sey4       AAATAG   666
sey1       AAATAG   666
sey6       AAATAG   666
sey2       AAATAG   666
sey7       AAATAG   666
sey5       AAATAA   666
sey3       AAATAG   666
           *****
```

Figure S1. Alignment of amino acid (**a**) and nucleotide (**b**) sequences of SEY (sey1-sey7). Asterisk indicates identical amino acid or nucleotide. Amino acids that are different from those of SEY1 are marked in yellow.

(a)

```
SC761-coaVa-ST121      MILGEFEVKYLTGFILILLLEGILTNSASAIEYSDLHHKSKLNSKRLSNAKMSFINPTQL     60
SC1133-coaVa-ST121     MILGEFEVKYLTGFILILLLEGILTNSASAIEYSDLHHKSKLNSKRLSNAKMSFINPTQL     60
SC1137-coaVa-ST121     MILGEFEVKYLTGFILILLLEGILTNSASAIEYSDLHHKSKLNSKRLSNAKMSFINPTQL     60
SC732-coaVa-ST121      MILGEFEVKYLTGFILILLLEGILTNSASAIEYSDLHHKSKLNSKRLSNAKMSFINPTQL     60
SC533-coaVIIb-ST45     MILGEFEVKYLTGFILILLLEGILTNSASAIAYSDLHHKSKFNSKRLSNAKMSFANPTDL     60
SC465-coaIIa-ST764     --MGEFEVKYLTGFILILLLEGIFTNSASAIEYSDLHHKSKFDSKRLSNAKMSFINPTQL     58
SC792-coaIIa-ST5       --MGEFEVKYLTGFILILLLEGIFTNSASAIEYSDLHHKSKFDSKRLSNAKMSFINPTQL     58
SC936-coaIIa-ST5       --MGEFEVKYLTGFILILLLEGIFTNSASAIEYSDLHHKSKFDSKRLSNAKMSFINPTQL     58
SC955-coaIIa-ST5       --MGEFEVKYLTGFILILLLEGIFTNSASAIEYSDLHHKSKFDSKRLSNAKMSFINPTQL     58
SC1096-coaVb-ST5425    MILGEFEVKYLTGFILILLLEGIFTNSASAIEYSDLHHKSKFDSKRLSNAKMSFINPTQL     60
SC519-coaIVa-ST30      --MGEFEVKYLTGFILILLLEGIFTNSASAIEYSDLHHKSKLDSKRLYNAKVSFANPTDL     58
SC1130-coaIIIa-ST8     --MGEFEVKYLTGFILILLLEGIFTNSASAIEYSDLHHKSKFDSKRLSNAKMSFINPTQL     58
SC526-coaIIIa-ST8      --MGEFEVKYLTGFILILLLEGIFTNSASAIEYSDLHHKSKFDSKRLSNAKMSFINPTQL     58
SC625-coaIIIa-ST8      --MGEFEVKYLTGFILILLLEGIFTNSASAIEYSDLHHKSKFDSKRLSNAKMSFINPTQL     58
SC546-coaIIIa-ST8      --MGEFEVKYLTGFILILLLEGIFTNSASAIEYSDLHHKSKFDSKRLSNAKMSFINPTQL     58
SC1019-coaIIIa-ST8     --MGEFEVKYLTGFILILLLEGIFTNSASAIEYSDLHHKSKFDSKRLSNAKMSFINPTQL     58
SC635-coaVIIa-ST1      --MGEFEVKYLTGFILILLLEGIFTNSASAIEYSDLHHKSKFDSKRLSNAKMSFINPTQL     58
SC458-coaVIIa-ST1      --MGEFEVKYLTGFILILLLEEILTNSASAIEYSDLHHKSKFDSKRLSNAKMSFINPTQL     58
SC470-coaVIIa-ST1      --MGEFEVKYLTGFILILLLEEILTNSASAIEYSDLHHKSKFDSKRLSNAKMSFINPTQL     58
SC560-coaVIIa-ST1      --MGEFEVKYLTGFILILLLEEILTNSASAIEYSDLHHKSKFDSKRLSNAKMSFINPTQL     58
SC735-coaIa-ST89       --MGEFEVKYLTGFILILLLEGIFTNSASAIEYSDLHHKSELDSKRLYNAKVSFANPTQL     58
SC756-coaIa-ST89       --MGEFEVKYLTGFILILLLEGIFTNSASAIEYSDLHHKSELDSKRLYNAKVSFANPTQL     58
SC778-coaIa-ST89       --MGEFEVKYLTGFILILLLEGIFTNSASAIEYSDLHHKSELDSKRLYNAKVSFANPTQL     58
SC912-coaVIIb-ST45     --MGEFEVKYLTGFILILLLEGIFTNSASAIEYSDLHHKSKLDSKRLYNAKVSFANPTDL     58
                         :****************** *:******* ********:::**** ***:** ***:*


SC761-coaVa-ST121      ENKKTNDRLLTHDLLFHDMFLNDASKKDFKVEFENEALSNEFINKNIDIYAGSYSYECHG     120
SC1133-coaVa-ST121     ENKKTNDRLLTHDLLFHDMFLNDASKKDFKVEFENEALSNEFINKNIDIYAGSYSYECHG     120
SC1137-coaVa-ST121     ENKKTNDRLLTHDLLFHDMFLNDASKKDFKVEFENEALSNEFINKNIDIYAGSYSYECHG     120
SC732-coaVa-ST121      ENKKTNDRLLTHDLLFHDMFLNDASKKDFKVEFENEALSNEFINKNIDIYAGSYSYECHG     120
SC533-coaVIIb-ST45     ENKKTNDRLLTHDLLFHDMFLNDAWKKDFKVEFENEALSNEFINKNIDIYTGSYSYECHG     120
SC465-coaIIa-ST764     ENKNTNDRLLKHDLLFHDMFVNVASKKDFKVEFENEALSKKFINKNIDIYAGSYSYECHG     118
SC792-coaIIa-ST5       ENKNTNDRLLKHDLLFHDMFVNVASKKDFKVEFENEALSKKFINKNIDIYAGSYSYECHG     118
SC936-coaIIa-ST5       ENKNTNDRLLKHDLLFHDMFVNVASKKDFKVEFENEALSKKFINKNIDIYAGSYSYECHG     118
SC955-coaIIa-ST5       ENKNTNDRLLKHDLLFHDMFVNVASKKDFKVEFENEALSKKFINKNIDIYAGSYSYECHG     118
SC1096-coaVb-ST5425    ENKNTNDRLLKHDLLFHDMFVNVASKKDFKVEFENEALSKKFINKNIDIYAGSYSYECHG     120
SC519-coaIVa-ST30      ENKKTNDRLLKHDLLFHDMFLNDAWKKDLKVEFENEALSKKFINKNIDVYAGSYSYECHG     118
SC1130-coaIIIa-ST8     ENKNTNDRLLKHDLLFHDMFVNDDWKKDFKVEFENEALSKKFINKDIDIFAGNYGYGCHG     118
SC526-coaIIIa-ST8      ENKNTNDRLLKHDLLFHDMFVNDDWKKDFKVEFENEALSKKFINKDIDIFAGNYGYGCHG     118
SC625-coaIIIa-ST8      ENKNTNDRLLKHDLLFHDMFVNDDWKKDFKVEFENEALSKKFINKDIDIFAGNYGYGCHG     118
SC546-coaIIIa-ST8      ENKNTNDRLLKHDLLFHDMFVNDDWKKDFKVEFENEALSKKFINKDIDIFAGNYGYGCHG     118
SC1019-coaIIIa-ST8     ENKNTNDRLLKHDLLFHDMFVNDDWKKDFKVEFENEALSKKFINKDIDIFAGNYGYGCHG     118
SC635-coaVIIa-ST1      ENKNTNDRLLKHDLLFHDMFVNDDWKKDFKVEFENEALSKKFINKDIDIFAGNYGYGCHG     118
SC458-coaVIIa-ST1      ENKNTNDRLLKHDLLFHDMFVNDDWKKDFKVEFENEALSKKFINKDIDIFAGNYGYGCHG     118
SC470-coaVIIa-ST1      ENKNTNDRLLKHDLLFHDMFVNDDWKKDFKVEFENEALSKKFINKDIDIFAGNYGYGCHG     118
SC560-coaVIIa-ST1      ENKNTNDRLLKHDLLFHDMFVNDDWKKDFKVEFENEALSKKFINKDIDIFAGNYGYGCHG     118
SC735-coaIa-ST89       ENKKTNDRLLKHDLLFHDMFVNDAWKKDFKVEFENEALSKKFVNKDIDIFAGNYGYGCHG     118
SC756-coaIa-ST89       ENKKTNDRLLKHDLLFHDMFVNDAWKKDFKVEFENEALSKKFVNKDIDIFAGNYGYGCHG     118
SC778-coaIa-ST89       ENKKTNDRLLKHDLLFHDMFVNDAWKKDFKVEFENEALSKKFVNKDIDIFAGNYGYGCHG     118
SC912-coaVIIb-ST45     ENKKTNDRLLKHDLLFHDMFVNDDWKKDFKVEFENEALSKKFINKDIDIFAGNYGYGCHG     118
                       ***:******.*********:*   ***:**********::*:**:**:::*.*.* ***
```

```
SC761-coaVa-ST121       GETNKTQCSYGGVTLSDNNKYDDYKNIPCNLWIDGHQTEIELTAVKTKKKIVTIQELDVQ    180
SC1133-coaVa-ST121      GETNKTQCSYGGVTLSDNNKYDDYKNIPCNLWIDGHQTEIELTAVKTKKKIVTIQELDVQ    180
SC1137-coaVa-ST121      GETNKTQCSYGGVTLSDNNKYDDYKNIPCNLWIDGHQTEIELTAVKTKKKIVTIQELDVQ    180
SC732-coaVa-ST121       GETNKTQCSYGGVTLSDNNKYDDYKNIPCNLWIDGHQTEIELTAVKTKKKIVTIQELDVQ    180
SC533-coaVIIb-ST45      GATNKTQCSYGGVTLSDNNKYDDYKNIPCNLWIDGHQTEKELTAVKTKKKIVTIQELDVQ    180
SC465-coaIIa-ST764      GATNKTQCSYGGVTLSDNNK----------------------------------------    138
SC792-coaIIa-ST5        GATNKTQCSYGGVTLSDNNK----------------------------------------    138
SC936-coaIIa-ST5        GATNKTQCSYGGVTLSDNNK----------------------------------------    138
SC955-coaIIa-ST5        GATNKTQCSYGGVTLSDNNK----------------------------------------    138
SC1096-coaVb- ST5425    GATNKTQCSYGGVTLSDNNK----------------------------------------    140
SC519-coaIVa-ST30       GETNKTQCSYGGVTLSDNNKYDDYKNIPCNLWIDGHQTEIELTAVKTKK-----------    167
SC1130-coaIIIa-ST8      GATNKTQCSYGGVTLSDNNKYDDYKNIPCNLWIDGHQTEIELTAVKTKKKIVTIQELEVQ    178
SC526-coaIIIa-ST8       GATNKTQCSYGGVTLSDNNKYDDYKNIPCNLWIDGHQTEIELTAVKTKKKIVTIQELEVQ    178
SC625-coaIIIa-ST8       GATNKTQCSYGGVTLSDNNKYDDYKNIPCNLWIDGHQTEIELTAVKTKKKIVTIQELEVQ    178
SC546-coaIIIa-ST8       GATNKTQCSYGGVTLSDNNKYDDYKNIPCNLWIDGHQTEIELTAVKTKKKIVTIQELEVQ    178
SC1019-coaIIIa-ST8      GATNKTQCSYGGVTLSDNNKYDDYKNIPCNLWIDGHQTEIELTAVKTKKKIVTIQELEVQ    178
SC635-coaVIIa-ST1       GATNKTQCSYGGVTLSDNNKYDDYKNIPCNLWIDGHQTEIELTAVKTKKKIVTIQELEVQ    178
SC458-coaVIIa-ST1       GATNKTQCSYGGVTLSDNNKYDDYKNIPCNLWIDGHQAEIELTAVKTKKKIVTIQELDVQ    178
SC470-coaVIIa-ST1       GATNKTQCSYGGVTLSDNNKYDDYKNIPCNLWIDGHQAEIELTAVKTKKKIVTIQELDVQ    178
SC560-coaVIIa-ST1       GATNKTQCSYGGVTLSDNNKYDDYKNIPCNLWIDGHQAEIELTAVKTKKKIVTIQELDVQ    178
SC735-coaIa-ST89        GETNKTLCSYGGVTLSDNNKYDDYKNIPCNLWIDGHQTGIELTAVKTKKKIVTIQELDVQ    178
SC756-coaIa-ST89        GETNKTLCSYGGVTLSDNNKYDDYKNIPCNLWIDGHQTGIELTAVKTKKKIVTIQELDVQ    178
SC778-coaIa-ST89        GETNKTLCSYGGVTLSDNNKYDDYKNIPCNLWIDGHQTGIELTAVKTKKKIVTIQELDVQ    178
SC912-coaVIIb-ST45      GETNKTQCSYGGVTLSDNNKYDNDKNIPCNLWIDGHQTEIELTAVKTKKKIVTIQELDVQ    178
                        * **** *************


SC761-coaVa-ST121       LRNYLSEKYKLYEKGGDIVKGYVKYHNDDEKNVEYDFYNLNGEYGHEVLKMYADNKTINS    240
SC1133-coaVa-ST121      LRNYLSEKYKLYEKGGDIVKGYVKYHNDDEKNVEYDFYNLNGEYGHEVLKMYADNKTINS    240
SC1137-coaVa-ST121      LRNYLSEKYKLYEKGGDIVKGYVKYHNDDEKNVEYDFYNLNGEYGHEVLKMYADNKTINS    240
SC732-coaVa-ST121       LRNYLSEKYKLYEKGGDIVKGYVKYHNDDEKNVEYDFYNLNGEYGHEVLKMYADNKTINS    240
SC533-coaVIIb-ST45      LRNYLNEKYKLYEKGGDIVKGYVKYHNDDEKNVEYDFYNLNGEYGHEVLKMYADNKTINS    240
SC465-coaIIa-ST764      ------------------------------------------------------------    138
SC792-coaIIa-ST5        ------------------------------------------------------------    138
SC936-coaIIa-ST5        ------------------------------------------------------------    138
SC955-coaIIa-ST5        ------------------------------------------------------------    138
SC1096-coaVb- ST5425    ------------------------------------------------------------    140
SC519-coaIVa-ST30       ------------------------------------------------------------    167
SC1130-coaIIIa-ST8      LRNYLNEKYKLYEQGGDIVKGYVKYYNDDEQNVEYDFYNLNGEYGREVLKMYADNKTINS    238
SC526-coaIIIa-ST8       LRNYLNEKYKLYEQGGDIVKGYVKYYNDDEQNVEYDFYNLNGEYGREVLKMYADNKTINS    238
SC625-coaIIIa-ST8       LRNYLNEKYKLYEQGGDIVKGYVKYYNDDEQNVEYDFYNLNGEYGREVLKMYADNKTINS    238
SC546-coaIIIa-ST8       LRNYLNEKYKLYEQGGDIVKGYVKYYNDDEQNVEYDFYNLNGEYGREVLKMYADNKTINS    238
SC1019-coaIIIa-ST8      LRNYLNEKYKLYEQGGDIVKGYVKYYNDDEQNVEYDFYNLNGEYGREVLKMYADNKTINS    238
SC635-coaVIIa-ST1       LRNYLNEKYKLYEQGGDIVKGYVKYYNDDEQNVEYDFYNLNGEYGREVLKMYADNKTINS    238
SC458-coaVIIa-ST1       LRNYLNEKYKLYEQGGDIVKGYVKYHNDDEQNIEYNFYNLNGEYGYEVLKMYADNKTINS    238
SC470-coaVIIa-ST1       LRNYLNEKYKLYEQGGDIVKGYVKYHNDDEQNIEYNFYNLNGEYGYEVLKMYADNKTINS    238
SC560-coaVIIa-ST1       LRNYLNEKYKLYEQGGDIVKGYVKYHNDDEQNIEYNSYNLNGEYGYEVLKMYADNKTINS    238
SC735-coaIa-ST89        LRNYLNEKYKLYELGGDIVKGYVKYHNDDEKNVEYDFYNLNGEYGHEVLKMYADNKTINS    238
SC756-coaIa-ST89        LRNYLNEKYKLYELGGDIVKGYVKYHNDDEKNVEYDFYNLNGEYGHEVLKMYADNKTINS    238
SC778-coaIa-ST89        LRNYLNEKYKLYELGGDIVKGYVKYHNDDEKNVEYDFYNLNGEYGHEVLKMYADNKTINS    238
SC912-coaVIIb-ST45      LRNYLNEKYKLYEQGGDIVKGYVKYHNDDEKNVEYDFYNLNGEYGHEVLKMYADNKTINS    238
```

```
SC761-coaVa-ST121        DKLHLDIYLFKS  252
SC1133-coaVa-ST121       DKLHLDIYLFKS  252
SC1137-coaVa-ST121       DKLHLDIYLFKS  252
SC732-coaVa-ST121        DKLHLDIYLFKS  252
SC533-coaVIIb-ST45       DKLHLDIYLFKS  252
SC465-coaIIa-ST764       ------------  138
SC792-coaIIa-ST5         ------------  138
SC936-coaIIa-ST5         ------------  138
SC955-coaIIa-ST5         ------------  138
SC1096-coaVb-ST5425      ------------  140
SC519-coaIVa-ST30        ------------  167
SC1130-coaIIIa-ST8       DKLHLDIYLFKS  250
SC526-coaIIIa-ST8        DKLHLDIYLFKS  250
SC625-coaIIIa-ST8        DKLHLDIYLFKS  250
SC546-coaIIIa-ST8        DKLHLDIYLFKS  250
SC1019-coaIIIa-ST8       DKLHLDIYLFKS  250
SC635-coaVIIa-ST1        DKLHLDIYLFKS  250
SC458-coaVIIa-ST1        DKLHLDIYLFKS  250
SC470-coaVIIa-ST1        DKLHLDIYLFKS  250
SC560-coaVIIa-ST1        DKLHLDIYLFKS  250
SC735-coaIa-ST89         DKLHLDIYLFKS  250
SC756-coaIa-ST89         DKLHLDIYLFKS  250
SC778-coaIa-ST89         DKLHLDIYLFKS  250
SC912-coaVIIb-ST45       DKLHLDIYLFKS  250
```

(b)

```
SC761-selw7           MILGEFEVKYLTGFILILLLEGILTNSASAIEYSDLHHKSKLNSKRLSNAKMSFINPTQL  60
TD101-selw10          ------------------MLEEILTNSVSAIEYSDLHHKSKLNSKRLYNAKVSFANPTDL  42
K17-selw9             ------------------MLEGIFTNSASAIEYSDLHHKSKLNSKRLYNAKVSFANPTDL  42
SC465-selw4           --MGEFEVKYLTGFILILLLEGIFTNSASAIEYSDLHHKSKFDSKRLSNAKMSFINPTQL  58
N315-selw4            --MGEFEVKYLTGFILILLLEGIFTNSASAIEYSDLHHKSKFDSKRLSNAKMSFINPTQL  58
TD3-selw5             --MGEFEVKYLTGFILILLLEGIFTNSASAIEYSDLHHKSKFDSKRLSNAKMSFINPTQL  58
MW2-selw3             -----------------------MTNSASAIEYSDLHHKSKFDSKRLSNAKMSFINPTQL  37
TD21-selw3            ------------------MLEEILTNSASAIEYSDLHHKSKFDSKRLSNAKMSFINPTQL  42
SC458-selw3           --MGEFEVKYLTGFILILLLEEILTNSASAIEYSDLHHKSKFDSKRLSNAKMSFINPTQL  58
TD115-selw3           ------------------MLEEILTNSASAIEYSDLHHKSKFDSKRLSNAKMSFINPTQL  42
SC1130-selw2          --MGEFEVKYLTGFILILLLEGIFTNSASAIEYSDLHHKSKFDSKRLSNAKMSFINPTQL  58
COL-selw2             --MGEFEVKYLTGFILILLLEGIFTNSASAIEYSDLHHKSKFDSKRLSNAKMSFINPTQL  58
TD8-selw6             --MGEFEVKYLTGFILILLLEGIFTNSASAIEYSDLHHKSKFDSKRLSNAKMSFINPTQL  58
RF122-selw1c          --MGEFEVKYLTGFILILLLEGIFTNSASAIEYSDLHHKSKLDSKGLYNAKVSFANPTEL  58
SC519-selw8           --MGEFEVKYLTGFILILLLEGIFTNSASAIEYSDLHHKSKLDSKRLYNAKVSFANPTDL  58
TCH60-selw8           --MGEFEVKYLTGFILILLLEGIFTNSASAIEYSDLHHKSKLDSKRLYNAKVSFANPTDL  58
SC756-selw1a          --MGEFEVKYLTGFILILLLEGIFTNSASAIEYSDLHHKSELDSKRLYNAKVSFANPTQL  58
Tager104-selw1c       MILGEFEVKYLTGFILILLLEGIFTNSASAIEYSDLHHKSKLDSKGLYNAKVSFANPTEL  60
TD123-selw1b          --MGEFEVKYLTGFILILLLEGIFTNSASAIEYSDLHHKSKLDSKRLYNAKVSFANPTDL  58
ISU926-selw1d         --MGEFEVKYLTGFILILLLEGIFTNSASAIEYSDLHHKSKLDSKRLYNAKVSFANPTDL  58
                                             :***.************:::** * ***:** ***:*
```

```
SC761-selw7        ENKKTNDRLLTHDLLFHDMFLNDASKKDFKVEFENEALSNEFINKNIDIYAGSYSYECHG 120
TD101-selw10       ENKNTNDRLLKHDLLFHDMFVNVASKKDFKVEFENEALSNEFINKNIDIYAGNYGYGCHG 102
K17-selw9          ENKNTNDRLLKHDLLFHDMFVNVASKKDFKVEFENEALSKKFINKNIDIYAGSYSYECHG 102
SC465-selw4        ENKNTNDRLLKHDLLFHDMFVNVASKKDFKVEFENEALSKKFINKNIDIYAGSYSYECHG 118
N315-selw4         ENKNTNDRLLKHDLLFHDMFVNVASKKDFKVEFENEALSKKFINKNIDIYAGSYSYECHG 118
TD3-selw5          ENKNTNDRLLKHDLLFHDMFVNDDWKKDFKVEFENEALSKKFINKNIDIYAGSYSYECHG 118
MW2-selw3          ENKNTNDRLLKHDLLFHDMFVNDDWKKDFKVEFENEALSKKFINKDIDIFAGNYGYGCHG 97
TD21-selw3         ENKNTNDRLLKHDLLFHDMFVNDDWKKDFKVEFENEALSKKFINKDIDIFAGNYGYGCHG 102
SC458-selw3        ENKNTNDRLLKHDLLFHDMFVNDDWKKDFKVEFENEALSKKFINKDIDIFAGNYGYGCHG 118
TD115-selw3        ENKNTNDRLLKHDLLFHDMFVNDDWKKDFKVEFENEALSKKFINKDIDIFAGNYGYGCHG 102
SC1130-selw2       ENKNTNDRLLKHDLLFHDMFVNDDWKKDFKVEFENEALSKKFINKDIDIFAGNYGYGCHG 118
COL-selw2          ENKNTNDRLLKHDLLFHDMFVNDDWKKDFKVEFENEALSKKFINKDIDIFAGNYGYGCHG 118
TD8-selw6          ENKNTKDRLLKHDLLFHDMFVNDDWKKDFKVEFENEALSKKFINKDIDIFAGNYGYGCHG 118
RF122-selw1c       ENKKTNDRLLKHDLLFHDMFVNDDWKKDFKVEFENEALSNEYINKNIDIYAGNYGYECHG 118
SC519-selw8        ENKKTNDRLLKHDLLFHDMFLNDAWKKDLKVEFENEALSKKFINKNIDVYAGSYSYECHG 118
TCH60-selw8        ENKKTNDRLLKHDLLFHDMFLNDAWKKDLKVEFENEALSKKFINKNIDVYAGSYSYECHG 118
SC756-selw1a       ENKKTNDRLLKHDLLFHDMFVNDAWKKDFKVEFENEALSKKFVNKDIDIFAGNYGYGCHG 118
Tager104-selw1c    ENKKTNDRLLKHDLLFHDMFVNDDWKKDFKVEFENEALSKKFINKDIDIFAGNYGYGCHG 120
TD123-selw1b       ENKKTNDRLLKHDLLFHDMFVNDDWKKDFKVEFENEALSKKFINKDIDIFAGNYGYGCHG 118
ISU926-selw1d      ENKKTNDRLLKHDLLFHDMFVNDDWKKDFKVEFENEALSKKFINKDIDIFAGNYGYGCHG 118
                   ***:*:****.*********:*   ***:***********:::**:**::**.*.* ***

SC761-selw7        GETNKTQCSYGGVTLSDNNKYDDYKNIPCNLWIDGHQTEIELTAVKTKKKIVTIQELDVQ 180
TD101-selw10       GATNKTQCSYGGVTLSDNNKYDDYKNIPCNLWIDGHQTEIELTAVKTKKKIVTIQELEVQ 162
K17-selw9          GATNKTQCSYGGVTLSDNNK---------------------------------------- 122
SC465-selw4        GATNKTQCSYGGVTLSDNNK---------------------------------------- 138
N315-selw4         GATNKTQCSYGGVTLSDNNK---------------------------------------- 138
TD3-selw5          GATNKTQCSYGGVTLSDNNKYDDYKNIPCNLWIDGHQAEIELTAVKTKKKIVTIQELDVQ 178
MW2-selw3          GATNKTQCSYGGVTLSDNNKYDDYKNIPCNLWIDGHQAEIELTAVKTKKKIVTIQELDVQ 157
TD21-selw3         GATNKTQCSYGGVTLSDNNKYDDYKNIPCNLWIDGHQAEIELTAVKTKKKIVTIQELDVQ 162
SC458-selw3        GATNKTQCSYGGVTLSDNNKYDDYKNIPCNLWIDGHQAEIELTAVKTKKKIVTIQELDVQ 178
TD115-selw3        GATNKTQCSYGGVTLSDNNKYDDYKNIPCNLWIDGHQAEIELTAVKTKKKIVTIQELDVQ 162
SC1130-selw2       GATNKTQCSYGGVTLSDNNKYDDYKNIPCNLWIDGHQTEIELTAVKTKKKIVTIQELEVQ 178
COL-selw2          GATNKTQCSYGGVTLSDNNKYDDYKNIPCNLWIDGHQTEIELTAVKTKKKIVTIQELEVQ 178
TD8-selw6          GATNKTQCSYGGVTLSDNNKYDDYKNIPCNLWIDGHQAEIELTAVKTKKKIVTIQELDVQ 178
RF122-selw1c       GRNQ-------------------------------------------------------- 122
SC519-selw8        GETNKTQCSYGGVTLSDNNKYDDYKNIPCNLWIDGHQTEIELTAVKTKK----------- 167
TCH60-selw8        GETNKTQCSYGGVTLSDNNKYDDYKNIPCNLWIDGHQTEIELTAVKTKK----------- 167
SC756-selw1a       GETNKTLCSYGGVTLSDNNKYDDYKNIPCNLWIDGHQTGIELTAVKTKKKIVTIQELDVQ 178
Tager104-selw1c    GATNKTQCSYGGVTLSDNNKYDDYKNIPCNLWIDGHQTEIKLTAVKTKKKIVTIQELDVQ 180
TD123-selw1b       GETNKTQCSYGGVTLSDNNKYDNDKNIPCNLWIDGHQTEIELTAVKTKKKIVTIQELDVQ 178
ISU926-selw1d      GEANKTQCSYGGVTFSDNNKYDDYKNIPCNLWIDGHQTEIELTAVKTKKKIVTIQELDVQ 178
                   *  :
```

```
SC761-selw7          LRNYLSEKYKLYEKGGDIVKGYVKYHNDDEKNVEYDFYNLNGEYGHEVLKMYADNKTINS 240
TD101-selw10         LRNYLNEKYKLYEQGGDIVKGYVKYYNDDEQNVEYDFYNLNGEYGREVLKMYADNKTINS 222
K17-selw9            ------------------------------------------------------------ 122
SC465-selw4          ------------------------------------------------------------ 138
N315-selw4           ------------------------------------------------------------ 138
TD3-selw5            LRNYLNEKYKLYEQGGDIVKGYVKYHNDDEQNIEYNFYNLNGEYGYEVLKMYADNKTINS 238
MW2-selw3            LRNYLNEKYKLYEQGGDIVKGYVKYHNDDEQNIEYNFYNLNGEYGYEVLKMYADNKTINS 217
TD21-selw3           LRNYLNEKYKLYEQGGDIVKGYVKYHNDDEQNIEYNFYNLNGEYGYEILKMYADNKTINS 222
SC458-selw3          LRNYLNEKYKLYEQGGDIVKGYVKYHNDDEQNIEYNFYNLNGEYGYEVLKMYADNKTINS 238
TD115-selw3          LRNYLNEKYKLYEQGGDIVKGYVKYHNDDEQNIEYNFYNLNGEYGYEVLKMYADNKTINS 222
SC1130-selw2         LRNYLNEKYKLYEQGGDIVKGYVKYYNDDEQNVEYDFYNLNGEYGREVLKMYADNKTINS 238
COL-selw2            LRNYLNEKYKLYEQGGDIVKGYVKYYNDDEQNVEYDFYNLNGEYGREVLKMYADNKTINS 238
TD8-selw6            LRNYLNEKYKLYEQGGDIVKGYVKYYNDDEQNVEYDFYNLNGEYGREVLKMYADNKTINS 238
RF122-selw1c         ------------------------------------------------------------ 122
SC519-selw8          ------------------------------------------------------------ 167
TCH60-selw8          ------------------------------------------------------------ 167
SC756-selw1a         LRNYLNEKYKLYELGGDIVKGYVKYHNDDEKNVEYDFYNLNGEYGHEVLKMYADNKTINS 238
Tager104-selw1c      LRNYLNEKYKLYEQGGDIVKGYVKYHNDDEKNVEYDFYNLNGEYGHEVLKIYADNKTINS 240
TD123-selw1b         LRNYLNEKYKLYEQGGDIVKGYVKYHND-EKNVEYDFYNLNGEYGHEVLKMYADNKTINS 237
ISU926-selw1d        LRNYLNEKYKLYEQGGDIVKGYVKYHNDDEKNVEYDFYNLNGEYGHEVLKMYADNKTINS 238
```

```
SC761-selw7          DKLHLDIYLFKS    252
TD101-selw10         DKLHLDIYLFKS    234
K17-selw9            ------------    122
SC465-selw4          ------------    138
N315-selw4           ------------    138
TD3-selw5            DKLHLDIYLFKS    250
MW2-selw3            DKLHLDIYLFKS    229
TD21-selw3           DKLHLDIYLFKS    234
SC458-selw3          DKLHLDIYLFKS    250
TD115-selw3          DKLHLDIYLFKS    234
SC1130-selw2         DKLHLDIYLFKS    250
COL-selw2            DKLHLDIYLFKS    250
TD8-selw6            DKLHLDIYLFKS    250
RF122-selw1c         ------------    122
SC519-selw8          ------------    167
TCH60-selw8          ------------    167
SC756-selw1a         DKLHLDIYLFKS    250
Tager104-selw1c      DKLHLDIYLFKS    252
TD123-selw1b         DKLHLDIYLFKS    249
ISU926-selw1d        DKLHLDIYLFKS    250
```

Figure S2. Alignment of SElW amino acid sequences of all the MRSA isolates analyzed in the present study (**a**) and representative strains of all the *selw* phylogenetic groups (**b**). Asterisk indicates identical amino acid. Dash denotes gap.

```
SELX14               MFKKHHSKNSILLKSILSLGIIYSGIFGINSKADASIQDSSSVHDKQFQKVEEVPNNSEK     60
SELX6                MFKKHHSKNSILLKSILSLGIIYSGSFGIYSKAGASTQNSSSLQDKQFQKVEEVPNNSEK     60
SELX11               MFKKHHSKNSILLKSILSLGIIYSGSLGIYSKANASTQNSSSVHDKQLQKVEEVPNNSEK     60
SELX4                MFKKYDSKNSILLKSILSLGIIYGGTFGIYPKADASTQNSSSVQDKQFQKVEEVPNNSEK     60
SC533(SELX16)        MFKKHHSKNSIVLKSILSLGIIYGGTFGIYPKADASTQNSSSVQDKQFQKVEEVPNNSEK     60
TIGER104(SELX15)     MFKKYESKNSIVLKSILSLGIIYGGTFGIYPKADASTQNSSSVQDKQFQKVEEVPNNSEK     60
SELX5                MFKKNDSKNSILLKSILSLGIIYGGTFGIYPKADASTQNSPSVQDKQFQKVEEVPNNSEK     60
SELX9                MFKKNDSKNSIVLKSILSLGIIYGGTFGIYPKADASTQNSSSVQDKQLQKVEEVPNNSEK     60
SELX1                MFKKYDSKNSIVLKSILSLGIIYGGTFGIYPKADASTQNSSSVQDKQLQKVEEVPNNSEK     60
SELX2                MFKKYDSKNSIVLKSILSLGIIYGGTFGIYPKADASTQNSSSVQDKQLQKVEEVPNNSEK     60
SELX-BOV2            MFKKHHSKNSIVLKSILSLGIIYGGTFGIYPKADASTQNSSSVQDKQLQKVEEVPNNSEK     60
SELXOV               MFKKYDSKNSIVLKSILSLGIIYGGTFGIYPKADASTQNSSSVQDKQLQKVEEVPNNSEK     60
SELX7                MFKKYDSKNSIVLKSILSLGIIYGGTFGIYPKADASTQNSSSVQDKQLQKVEEVPNNSEK     60
SELX12               MFKKYDSKNSIVLKSILSLGIIYGGTFGIYPKADASTQNSSSVQDKQLQKVEEVPNNSEK     60
SELX10               MFKKYDSKNSIVLKSILSLGIIYGGTFGIYPKADASTQNSSSVQDKQFQKVEEVPNNSEK     60
SELX-BOV1            MFKKYDSKNSIVLKSILSLGIIYGGTFGIYPKADASTQNSSSVQDKQFQKVEEVPNNSEK     60
SELX3                MFKKYDSKNSIVLKSILSLGIIYGGTFGIYPKADASTQNSSSVQDKQLQKVEEVPNNSEK     60
SELX8                MFKKYDSKNSIVLKSILSLGIIYSGSFGIYPKADASTQNSSSVQDKQLQKVEEVPNNSEK     60
                     **** .*****:***********.* :**  **.** *:* *::***:************


SELX14               ALVKKLYDRYSQNTINGKSNKSRNWVYSERPLNENQVRIHLEGTYTVAGRVYTPKRNITL     120
SELX6                ALVKKLYDRYSQNTINGKSNKSRNWVYSERPLNENQVRIHLEGTYTVADRVYTPKRNITL     120
SELX11               ALVKKLYDRYSKNTINGKSNKSRNWVYSERPLNGNQVRIHLEGTYTVAGRVYTPKRNITL     120
SELX4                TLVKKLYDRYSKNTINGKSNKSRNWVYSERPLNENQVRIHLEGTYRVADRVYTPKRNITL     120
SC533(SELX16)        ALVKKLYDRYSKNTINGKSNKSRNWVYSERPLNGNQVRIHLEGTYTVAGRVYTPKRNITL     120
TIGER104(SELX15)     ALVKKLYDRYSQNTINGKSNKARNWVYSERPLNENQVRIHLEGTYTVAGRVYTPKRNITL     120
SELX5                ALVKKLYDRYSQNTINGKSNKSRNWVYSERPLNENQVRINLEGTYRVADRVYTPKRNITL     120
SELX9                ALVKKLYDRYSQNTINGKSNKSRNWVYSERPLNENQVRIHLEGTYRVADRVYTPKRNITL     120
SELX1                ALVKKLYDRYSKDTINGKSNKSRNWVYSERPLNENQVRIHLEGTYTVADRVYTPKRNITL     120
SELX2                ALVKKLYDRYSKDTINGKSNKSRNWVYSERPLNENQVRIHLEGTYTVAGRVYTPKRNITL     120
SELX-BOV2            ALVKKLYDRYSKDTINGKSNKSRNWVYSERPLNENQVRIHLEGTYRVADRVYTPKRNITL     120
SELXOV               ALVKKLYDRYSKDTINGKSNKSRNWVYSERPLNENQVRIHLEGTYRVADRVYTPKRNITL     120
SELX7                ALVKKLYDRYSKDTINGKSNKSRNWVYSERPLNENQVRIHLEGTYRVADRVYTPKRNITL     120
SELX12               ALVKKLYDRYSKDTINGKSNKSRNWVYSERPLNENQVRIHLEGTYRVADRVYTPKRNITL     120
SELX10               ALVKKLYDRYSQNTINGKSNKSRNWVYSERPLNENQVRIHLEGTYTVAGRVYTPKRNITL     120
SELX-BOV1            ALVKKLYDRYSQNTINGKSNKARNWVYSERPLNENQVRIHLEGTYRVADRVYTPKRNITL     120
SELX3                ALVKKLYDRYSKDTINGKSNKSRNWVYSERPLNENQVRIHLEGTYTVAGRVYTPKRNITL     120
SELX8                ALVKKLYDRYSKDTINGKSNKSRNWVYSERPLNENQVRIHLEGTYTVAGRVYTPKRNITL     120
                     :**********::********:*********** *****:***** **.***********


SELX14               NKEVVTLKELDHIVRFAHISYGLYMGEHLPKGNIVINTKDGGKYTLESHKELQKDRENVE     180
SELX6                NKEVITLKELDHIIRFAHISYGLYMGEHLPKGNIVINTKDGGKYTLESHKELQKDRENVK     180
SELX11               NKEVVTLKELDHIIRFAHISYGLYMGEHLPKGNIVINTKNGGKYTLESHKELQKDRENVK     180
SELX4                NKEVVTLKELNHIIRFAHISYGLYMGEHLSKGDIVINTKDGGKYTLESHKELQKNRENVK     180
SC533(SELX16)        NKEVVTLKELDHIIRFAHISYGLYMGEHLPKGNIVINTKNGGKYTLESHKELQKNRENVE     180
TIGER104(SELX15)     NKEVVTLKELDHIIRFAHISYGLYMGEHLPKGNIVINTKNGGKYTLESHKELQKNRENVE     180
SELX5                NKEVVTLKELDHIIRFAHISYGLYMGEHLPKGNIVINTKDGGKYTLESHKELQKDRENVK     180
SELX9                NKEIVTLKELDHIIRFAHISYGLYMGEHLPKGNIVINTKNGGKYTLESHKELQKDRENVK     180
SELX1                NKEVVTLKELDHIIRFAHISYGLYMGEHLPKGNIVINTKDGGKYTLESHKELQKDRENVK     180
SELX2                NKEVVTLKELDHIIRFAHISYGLYMGEHLPKGNIVINTKDGGKYTLESHKELQKDRENVK     180
SELX-BOV2            NKEVVTLKELDHIIRFAHISYGLYMGEHLPKGNIVINTKNGGKYTLESHKELQKDRENVK     180
SELXOV               NKEVVTLKELDHIIRFAHISYGLYMGEHLPKGNIVINTKNGGKYTLESHKELQKDRENVK     180
SELX7                NKEVITLKELDHIIRFAHISYGLYMGEHLPKGNIVINTKNGGKYTLESHKELQKDRENVK     180
SELX12               NKEVVTLKELDHIIRFAHISYGLYMGEHLPKGNIVINTKDGGKYTLESHKELQKDRENVK     180
SELX10               NKEVVTLKELDHIIRFAHISYGLYMGEHLPKGNIVINTKNGGKYTLESHKELQKDRENVK     180
SELX-BOV1            NKEVVTLKELDHIIRFAHISYGLYMGEHLPKGNIVINTKNGGKYTLESHKELQKNRENVE     180
SELX3                NKEVVTLKELDHIIRFAHISYGLYMGEHLPKGNIVINTKNGGKYTLESHKELQKNRENVE     180
SELX8                NKEVVTLKELDHIIRFAHISYGLYMGEHLPKGNIVINTKNGGKYTLESHKELQKNRENVE     180
                     ***::*****:**:*************** **:******:**************:****:
```

```
SELX14              INTADIKNVTFELVKSVNDIEQV    203
SELX6               INTADIKNVTFDLVKSVNDIEQV    203
SELX11              INTDDIKNVTFELVKSVNDIEQV    203
SELX4               INTADVKNVTFELVKSVNDIEQV    203
SC533(SELX16)       INTADVENVTFDLVKSVNDIEQV    203
TIGER104(SELX15)    INTADVENVTFDLVKSVNDIEQV    203
SELX5               INTADIKNVTFKLVKSVNDIEQV    203
SELX9               INTDDIKNVTFELVKSVNDIEQV    203
SELX1               INTADIKNVTFKLVKSVNDIEQV    203
SELX2               INTADIKNVTFKLVKSVNDIEQV    203
SELX-BOV2           INTDDIKNVTFELVKSVNDIEQV    203
SELXOV              NNTDDIKNVTFELVKSVNDIEQV    203
SELX7               INTDDIKNVTFELVKSVNDIEQV    203
SELX12              INTDDIKNVTFELVKSVNDIEQV    203
SELX10              IKTDDIKNVTFELVKRVNDIEQV    203
SELX-BOV1           INTDDIKNVTFELVKSVNDIEQV    203
SELX3               INTDDIKNVTFELVKSVNDIEQV    203
SELX8               INTDDIKNVTFELVKSVNDIEQV    203
                     :* *::****.*** *******
```

Figure S3. Alignment of SElX amino acid sequences representing subtypes SElX1-SElX16. Asterisk indicates identical amino acid. Among the 17 *selx* alleles described by Wilson et al [21], sequence data were available for 14 subtypes (*selx*1-*selx*14). Therefore, SElX sequences of TIGER104 and SC533 which were distinct from SElX1-SElX14 were tentatively assigned as SElX15 and SElX16. Putative cleavage site of signal peptide is shown by triangle. Sialic acid-binding region reported previously [22,23] is shown in yellow.

```
JKD6159-selz7        MRKVLILITLLFGYSCYLLLEAKAETQNDPNISELNKASQYTGSWHNIWYLYNSDPVKAK 60
M-selz8              MRKVFILITLLFGYSSYSLLEAKAETQNDPNISELNKSSQYTGSWHNIWYLYNSDPVNAK 60
SC532-selz6          MRKVFILITLLFGYSSYSLSEAKAETQNDLNISELNKSSQYTGSWHNIWYLYNSDPVNAK 60
NCTC6131-selz9       MRKVFILITLLFGYSSYSLLQAKAETQNDPNISELNKSSQYTGSWHNIWYLYNSDPVNAK 60
WA-MRSA-59-selz4     MRKVFILITLLFGYSSYSLLEARAETQNDPNISELNKSSQYTGSWHNIWYLYNSDPVNAK 60
MOK042-selz2         MRKIFILITLLFGYSSYSLLEARAETQNDPNISELNKSSQYTGSWHNIWYLYNSDPVNAK 60
15584-selz5          MRKVFILITLLFGYSSYSLLEARAETQNDPNISELNKSSQYTGSLHNIWYLYNSDPVNAK 60
SC457-selz1          MRKVFILITLLFGYSSYSLLEARAETQNDPNISELNKSSQYTGSWHNIWYLYNSDPVNAK 60
SC635-selz3          MRKVFILITLLFGYSSYSLLEARAETQNDPNISELNKSSQYTGSWHNIWYLYNSDPVNAK 60
                     ***::**********.* * :*:****** *******:****** ************:**

JKD6159-selz7        KTKLSDKFLSHDFIVPINSPGHYDYVKTELKDSTMASSFDGKEVDIFGVNYFHQCYFSNE 120
M-selz8              KIKLSDKFLSHEFIVPINNPSHYDYVKTELKDSTMASSFDGKEVDIFGVNYFDQCYFLNE 120
SC532-selz6          KIKLSDKFLSHDFIVPINSPGHYDYVKTELKDSTMASSFNGKEVDIFGVNYFDQCYFSNE 120
NCTC6131-selz9       KIKLSDKFLSHDFIVPINSPGHYDYVKTELKDSTMASSFDGKEVDIFGVNYFDQCYFSNE 120
WA-MRSA-59-selz4     KIKVSDKFLSHDFIVPINNPGHYDYVKTELKDSTMASSFDGKEVDIFGVNYFDQCYFSNE 120
MOK042-selz2         KIKLSDKFLSHDFIVPINNPGHYDYVKTELKDSTMASSFDGKEVDIFGVNYFDQCYFSNE 120
15584-selz5          KIKLSDKFLSHDFIVPINNPGHYDYVKTELKDSTMASSFDGKEVDIFGVNYFDQCYFSNE 120
SC457-selz1          KIKLSDKFLSHDFIVPINNPGHYDYVKTELKDSTMASSFDGKEVDIFGVNYFDQCYFSNE 120
SC635-selz3          KIKLSDKFLSHDFIVPINNPGHYDYVKTELKDSTMASSFDGKEVDIFGVNYFDQCYFSNE 120
                     * *:*******:******.*.*****************:************.**** **

JKD6159-selz7        NIQCDSNQGAGSKKTCMYGGITLNENNTNNRIQPIVVKVYENDSVTLSFDINIDKETVTI 180
M-selz8              NIQCDSNQGAGSKKTCMYGGITLNENNTNNRIQPIVVKVYENDSVTLSFDINIDKETVTI 180
SC532-selz6          NIQCDSNQGAGSKKTCMYGGITLNENNTNNRIQPIVVKVYENDSVTLSFDINIDKETVTI 180
NCTC6131-selz9       NIQCDSNQGAGSKKTCMYGGITLNENNTNNRIQPIVVKVYENDSVTLSFDINIDKETVTI 180
WA-MRSA-59-selz4     NIQCDSNQGAGSKKTCMYGGITLNENNTNNRIQPIVVKVYENDSVTLSFDINIDKETVTI 180
MOK042-selz2         NIQCDSNQGGGSKKTCMYGGITLNENNTNNRIQPIVVKVYENDSVTLSFDINIDKETVTI 180
15584-selz5          NIQCDSNQGAGSKKTCMYGGITLNENNTNNRIQPIVVKVYENDSVTLSFDINIDKETVTI 180
SC457-selz1          NIQCDSNQGGGSKKTCMYGGITLNENNTNNRIQPIVVKVYENDSVTLSFDINIDKETVTI 180
SC635-selz3          NIQCDSNQGAGSKKTCMYGGITLNENNTNNRIQPIVVKVYENDSVTLSFDINIDKETVTI 180
                     *********.**************************************************

JKD6159-selz7        QELDYKVRNKLISKINLYHLGGTSYETGYIKFIENANRYYWYDMMPDPSFTQSKYLMIYR 240
M-selz8              QELDYKVRNKLISKINLYHLGGTSYETGYIKFIENGNRYYWYDMMPDPGFTQSKYLMIYR 240
SC532-selz6          QELDYKVRNKLISKINLYHLGGTSYETGYIKFIENGNRYYWYDMMPDPGFTQSKYLMIYR 240
NCTC6131-selz9       QELDYKVRNKLISKINLYHLGGTSYETGYIKFIENGNRYYWYDMMPDPGFTQSKYLMIYR 240
WA-MRSA-59-selz4     QELDYKVRNKLISKINLYHLGGTSYETGYIKFIENGNRYYWYDMMPDPGFTQSKYLMIYR 240
MOK042-selz2         QELDYKVRNKLISKINLYHLGGTSYETGYIKFIENGNRYYWYDMMPDPGFTQSKYLMIYR 240
15584-selz5          QELDYKVRNKLISKINLYHLGGTSYETGYIKFIENGNRYYWYDMMPDPGFTQSKYLMIYR 240
SC457-selz1          QELDYKVRNKLISKINLYHLGGTSYETGYIKFIENGNRYYWYDMMPDPGFTQSKYLMIYR 240
SC635-selz3          QELDYKVRNKLISKINLYHLGGTSYETGYIKFIENGNRYYWYDMMPDPGFTQSKYLMIYR 240
                     ***********************************.************.***********

JKD6159-selz7        GNETVESAKTEIEVHLTKK     259
M-selz8              GNETVESAKTEIEVHLTKK     259
SC532-selz6          GNETVESAKTEIEVHLTKK     259
NCTC6131-selz9       GNETVESAKTEIEVHLTKK     259
WA-MRSA-59-selz4     GNETVESAKTEIEVHLTKK     259
MOK042-selz2         GNETVESAKTEIEVHLTKK     259
15584-selz5          GNETVESAKTEIEVHLTKK     259
SC457-selz1          GNETVESAKTEIEVHLTKK     259
SC635-selz3          GNETVESAKTEIEVHLTKK     259
                     *******************
```

Figure S4. Alignment of SElZ amino acid sequences representing subtypes SElZ1-SElZ9. Asterisk indicates identical amino acid.

Table S1. Nucleotide Sequence identities (percentage) of *sey* gene among selected *S. arueus* isolates and those of reported *S. argenteus* strain.

Strain	species	genotype	*sey* subtype	Identity with strain					
				SC823	SC533	SC1097	K5	RF122	SG01
SC612	*S. aureus*	*coa*Ia-ST89	sey5	99.7	99.5	99.1	99.4	99.2	98.8
SC823	*S. aureus*	*coa*IIa-ST764	sey3		99.2	98.8	99.1	98.9	98.5
SC533	*S. aureus*	*coa*VIIb-ST45	sey2			99.2	99.2	99.7	98.9
SC1097	*S. aureus*	*coa*IIa-ST5	sey1				98.8	98.9	98.5
K5 (CP020656)	*S. aureus*		sey6					98.8	98.2
RF122 (AJ938182)	*S. aureus*		sey7						98.6
SG01 (MN166481)	*S. argenteus*	*coa*XId, ST2250							

Table S2. Nucleotide Sequence identities (percentage) of *selw* gene among selected *S. arueus* isolates and those of reported *S. argenteus* strains.

Strain	species	genotype	*selw* group	Identity with strain								
				SC635	SC560	SC465	SC761	SC519	SC756	SC912	TD13	SG03
SC635	*S. aureus*	*coa*VIIa-ST1	group 2		97.8	97.5	95.4	94.9	96.2	96.4	93.9	94.0
SC560	*S. aureus*	*coa*VIIa-ST1	group 3			97.0	95.2	94.7	96.0	95.8	93.2	93.3
SC465	*S. aureus*	*coa*IIa-ST764	group 4				96.3	95.6	95.2	94.7	92.9	93.0
SC761	*S. aureus*	*coa*Va-ST121	group 7					96.2	95.0	95.8	92.9	93.1
SC519	*S. aureus*	*coa*IVa-ST30	group 8						96.9	96.8	94.2	94.4
SC756	*S. aureus*	*coa*Ia-ST89	group 1a							97.2	94.7	94.9
SC912	*S. aureus*	*coa*VIIb-ST45	group 1b								95.0	95.3
TD13 (MH484208)	*S. argenteus*	*coa*XId, ST2854										99.9
SG03 (MN159013)	*S. argenteus*	*coa*XV-ST1223										

Table S3. Nucleotide Sequence identities (percentage) of *selx* gene among selected *S. arueus* isolates and those of reported *S. argenteus* strain.

Strain	species	genotype	*selx* subtype	Identity with strain						
				SC711	SC717	SC838	SC460	SC533	selX8	SG02
SC657	*S. aureus*	*coa*VIIa-ST1	selx1	97.9	98.7	96.1	94.1	94.3	96.7	94.1
SC711	*S. aureus*	*coa*VIIa-ST1	selx5		96.6	95.6	94.1	94.0	94.8	92.6
SC717	*S. aureus*	*coa*IIIa-ST8	selx2			96.4	95.6	94.6	97.1	94.1
SC838	*S. aureus*	*coa*Va-ST121	selx10				94.5	96.5	96.9	93.3
SC460	*S. aureus*	*coa*Ia-ST89	selx6					94.3	95.4	92.8
SC533	*S. aureus*	*coa*VIIb-ST45	selx16						95.4	92.0
selX8 (HQ850970)	*S. aureus*		selx8							94.3
SG02 (MN158996)	*S. argenteus*	*coa*XIV-ST2198								

Table S4. Nucleotide Sequence identities (percentage) of *selz* gene among selected *S. arueus* isolates and those of reported *S. argenteus* strain.

Strain	species	genotype	*selz* subtype	Identity with strain			
				SC635	SC561	Tokyo 12480	SG01
SC485	*S.aureus*	*coa*IIa-ST764	selz1	99.5	98.5	96.5	99.6
SC635	*S.aureus*	*coa*VIIa-ST12	selz3		98.7	96.8	99.6
SC561	*S.aureus*	*coa*Ia-ST89	selz6			96.5	98.3
Tokyo 12480 (AP019712)	*S.aureus*		selz7				96.2
SG01 (MN166481)	*S.argenteus*	*coa*XId, ST2250					

Table S5. Primers used in the present study.

**Target gene**	**Primer**	**Nucleotide sequence**	**Product size**	**Aim**	**Reference**
selx	selx-1	(+) AGCAGACGCGTCAACACAAA	513 bp (+selx-2)	Detection	Wilson et al., 2011
	selx-2	(-) ACTTGTTCAATGTCATTAACACTTTTCAC		Detection	
	selx-F1	(+) GTCCAATTATGTGTAGACGA	ca. 900 bp (+selx-R1)	Sequencing	Aung et al., 2019
	selx-R1	(-) GAATTTTCTATATGATGGTGCT		Sequencing	
sey	sey-F	(+) CAATGTACGGACAGTGCTCTACAA	189 bp (+sey-R)	Detection	Ono et al., 2015
	sey-R	(-) TGACCGTTAACAAACAAGTTCATTC		Detection	
	sey-1	(+) GAAATATTGATATATAGATCATT	843 bp (+sey-2)	Sequencing	Aung et al., 2019
	sey-2	(-) CCTAAGAACTTAATKTCCTAAGC		Sequencing	
selw	selw-F2	(+) GGAATTTTTACAAATTCAGCG	323bp (+selw-R2)	Detection	Aung et al., 2019
	selw-R2	(-) CTACATTGCGTTTTATTGGTTG		Detection	
	selw-F3	(+) TTGTTTTGGGGGAGTTTGAAG	ca.800 bp (+selw-R4/R3)	Sequencing	
	selw-R4	(-) ACTTTATATTTCACTGTCAAAG		Sequencing	
	selw-R3	(-) GTCAAAGATTATTAATGATTAAC		Sequencing	
selz	selz-F1	(+) GGTTACAGTAGCTATTCTTTGTTG	ca.500 bp (+selz-R1)	Detection	Aung et al., 2019
	selz-R1	(-) GTAAACTTTTACAACAATAGGCTG		Detection	
	selz-F2	(+) GTTATAAAATAGCAATGGTTG	ca.1 kb (+selz-R2)	Sequencing	
	selz-R2	(-) GAGCAACTTTTCCAAGTCGC		Sequencing	
	selz-R3	(-) CATTTGAAAAATAGCATTGATC		Sequencing	
	selz-R4	(-) GATAAGTTGCTCTATGTCTA	ca.800 bp (+selz-F2)	Sequencing	
sel26	sel26-F1	(+) CTTATGCTGATGTAGGTGTTC	ca. 300 bp (+sel27-R1)	Detection	Aung et al., 2019
	sel26-R1	(-) CATTTATCCAAAGATTTATCGG		Detection	
	sel26-5p-F1	(+) GAAGAAAAACACGATAACGGTG	ca.1.4 kb (+sel28-3p-R1)	Sequencing	
sel27	sel27-F1	(+) TGAAGGCGCTCTATGAATCAG	ca. 500bp (+sel28-R1)	Detection	Aung et al., 2019
	sel27-R1	(-) TACTCTGCGTAAAATTTGGG		Detection	
	sel27-3p-R1	(-) ACAGGCTATCGTAAATCGCT		Sequencing	

Table 6. GenBank accession numbers assigned to *selx*, *sey*, *selw* and *selz* detected in the present study.

selx			***sey***			***selw***			***selz***		
Specimen ID	genotype	**Accession No**	**Specimen ID**	**genotype**	**Accession No**	**Specimen ID**	**genotype**	**Accession No**	**Specimen ID**	**genotype**	Accession No
SC460	*coa* Ia-ST89	MN257075	SC612	*coa* Ia-ST89	MN257099	SC735	*coa* Ia-ST89	MN257123	SC532	*coa* Ia-ST89	MN257147
SC654	*coa* Ia-ST89	MN257076	SC575	*coa* Ia-ST89	MN257100	SC756	*coa* Ia-ST89	MN257124	SC561	*coa* Ia-ST89	MN257148
SC735	*coa* Ia-ST89	MN257077	SC588	*coa* Ia-ST89	MN257101	SC778	*coa* Ia-ST89	MN257125	SC1103	*coa* Ia-ST89	MN257149
SC698	*coa* IIa-ST5	MN257078	SC823	*coa* IIa-ST5	MN257102	SC465	*coa* IIa-ST764	MN257126	SC485	*coa* IIa-ST764	MN257150
SC714	*coa* IIa-ST764	MN257079	SC610	*coa* IIa-ST764	MN257103	SC792	*coa* IIa-ST5	MN257127	SC585	*coa* IIa-ST5	MN257151
SC1100	*coa* IIa-ST5	MN257080	SC1097	*coa* IIa-ST5	MN257104	SC936	*coa* IIa-ST5	MN257128	SC1097	*coa* IIa-ST5	MN257152
SC825	*coa* IIa-ST764	MN257081	SC630	*coa* IIa-ST5	MN257105	SC955	*coa* IIa-ST5	MN257129	SC457	*coa* Va-ST121	MN257153
SC709	*coa* IIa-ST764	MN257082	SC1100	*coa* IIa-ST764	MN257106	SC526	*coa* IIIa-ST8	MN257130	SC732	*coa* Va-ST121	MN257154
SC822	*coa* IIa-ST5	MN257083	SC582	*coa* IIIa-ST8	MN257107	SC546	*coa* IIIa-ST8	MN257131	SC703	*coa* Va-ST121	MN257155
SC978	*coa* IIIa-ST8	MN257084	SC569	*coa* IIIa-ST8	MN257108	SC625	*coa* IIIa-ST8	MN257132	SC595	*coa* Va-ST121	MN257156
SC699	*coa* IIIa-ST8	MN257085	SC625	*coa* IIIa-ST8	MN257109	SC1019	*coa* IIIa-ST8	MN257133	SC584	*coa* Va-ST121	MN257157
SC706	*coa* IIIa-ST8	MN257086	SC628	*coa* IIIa-ST8	MN257110	SC1130	*coa* IIIa-ST8	MN257134	SC568	*coa* Va-ST121	MN257158
SC526	*coa* IIIa-ST8	MN257087	SC633	*coa* IIIa-ST8	MN257111	SC519	*coa* IVa-ST30	MN257135	SC462	*coa* Va-ST121	MN257159
SC1019	*coa* IIIa-ST8	MN257088	SC910	*coa* Va-ST121	MN257112	SC761	*coa* Va-ST121	MN257136	SC635	*coa* VIIa-ST12	MN257160
SC1060	*coa* IIIa-ST8	MN257089	SC736	*coa* Va-ST121	MN257113	SC732	*coa* Va-ST121	MN257137			
SC1137	*coa* Va-ST121	MN257090	SC1133	*coa* Va-ST121	MN257114	SC1133	*coa* Va-ST121	MN257138			
SC798	*coa* Va-ST121	MN257091	SC798	*coa* Va-ST121	MN257115	SC1137	*coa* Va-ST121	MN257139			
SC910	*coa* Va-ST121	MN257092	SC578	*coa* Va-ST121	MN257116	SC1096	*coa* Vb-ST5425 (ST5 SLV)	MN257140			
SC1096	*coa* Vb-ST5425 (ST5 SLV)	MN257093	SC635	*coa* VIIa-ST12	MN257117	SC458	*coa* VIIa-ST1	MN257141			
SC958	*coa* VIIa-ST1	MN257094	SC598	*coa* VIIa-ST1	MN257118	SC470	*coa* VIIa-ST1	MN257142			
SC1065	*coa* VIIa-ST1	MN257095	SC634	*coa* VIIa-ST1	MN257119	SC560	*coa* VIIa-ST1	MN257143			
SC1071	*coa* VIIa-ST1	MN257096	SC626	*coa* VIIa-ST1	MN257120	SC635	*coa* VIIa-ST12	MN257144			
SC650	*coa* VIIa-ST1	MN257097	SC614	*coa* VIIa-ST1	MN257121	SC533	*coa* VIIb-ST45	MN257145			
SC533	*coa* VIIb-ST45	MN257098	SC533	*coa* VIIb-ST45	MN257122	SC912	*coa* VIIb-ST45	MN257146			
